# An oral heme oxygenase inhibitor targets immunosuppressive perivascular macrophages in preclinical models of cancer

**DOI:** 10.1126/scitranslmed.ads3085

**Published:** 2025-08-06

**Authors:** Meriem Bahri, Taha Al-Adhami, Emre Demirel, Jit Sarkar, Karen T. Feehan, Joanne E. Anstee, Tik Shing Cheung, Dominika Sosnowska, Chloé A. Woodman, William Macmorland, Dorothy D. Yang, James Rosekilly, Renee Gitsaki-Taylor, Cheryl E. Gillett, Cheryl L. Scudamore, James Spicer, Khondaker Miraz Rahman, James N. Arnold

**Affiliations:** 1 School of Cancer and Pharmaceutical Sciences, King’s College London, London, SE1 1UL, United Kingdom; 2 Institute of Pharmaceutical Sciences, King’s College London, London, SE1 9NH, United Kingdom; 3 ExePathology, Exmouth, EX81TN, United Kingdom

**Keywords:** Heme oxygenase-1, immunotherapy, macrophages, microenvironment, perivascular, tumor

## Abstract

A subset of perivascular tumor-associated macrophages (PvTAMs), marked by the expression of the lymphatic vessel endothelial hyaluronan receptor-1 (LYVE-1), has previously been identified. These macrophages support the maintenance of an immunologically ‘cold’ tumor microenvironment (TME) through their expression of the enzyme heme oxygenase-1 (HO-1). To date, no HO inhibitors are in clinical development for the treatment of cancer. In this study, we develop and characterize an orally bioavailable HO inhibitor, KCL-HO-1i. In the spontaneous murine *MMTV-PyMT* model of breast cancer, targeting PvTAM function with KCL-HO-1i synergizes with the immune-stimulatory properties of chemotherapy drugs to deliver durable immunological control of tumor growth. This synergy is achieved by creating an immunologically ‘hot’ TME, characterized by an increased prevalence of CD8^+^ T-cells with effector function. This study identifies KCL-HO-1i as a non-toxic, orally bioavailable small molecule immunotherapeutic for targeting the function of a key subset of pro-tumoral TAMs in cancer.

## Introduction

Macrophages are an abundant immune cell type within the tumor microenvironment (TME), which support a variety of pro-tumoral processes (1–9). Due to their plasticity, they represent a phenotypically heterogeneous population of cells which develop specialized functions within the TME (9–11). Considering their tumor-promoting properties, tumor associated macrophages (TAMs) are a therapeutic target in cancer. However, targeting TAMs collectively using pan-depleting approaches such as neutralizing the colony stimulating factor (CSF)-1 and CSF-1 Receptor (CSF-1R) axis which supports TAM chemotaxis, differentiation and survival (8, 12–14), has unfortunately delivered limited clinical efficacy as a single agent therapy (15–17). As both anti- and pro-tumoral TAM subsets co-exist within a single TME (18, 19), more selective therapeutic approaches which target key pro-tumoral TAM subsets, while preserving the biological function of anti-tumoral subsets, may provide superior therapeutic responses.

Perivascular TAMs (PvTAMs) reside in close proximity to blood vasculature (<15-20 µm) (20, 21) and support a variety of pro-tumoral functions including neo-angiogenesis (18, 21), metastasis (3, 22, 23), immune exclusion (9), and resistance to (9) and recurrence following (24) chemotherapy treatment. PvTAMs have been reported to be relatively resistant to CSF1-CSF1R blockade (25, 26), highlighting the need for novel therapeutic strategies for targeting this population. We recently characterized a subset of PvTAMs in the mouse mammary tumor virus promoter driven polyoma middle T-antigen (*MMTV-PyMT*) spontaneous murine model of breast cancer that express the lymphatic vessel endothelial hyaluronan receptor-1 (LYVE-1^+^)MHCII^lo^CD206^hi^ and are polarized by IL-6 within the TME (9, 18). LYVE-1^+^ PvTAMs form CCR5-dependent collaborative multicellular nest structures in the perivascular (Pv) niche and maintain an immunologically cold TME through their expression of the enzyme heme oxygenase-1 (HO-1) (9). HO-1 represents an immunotherapy target for the immunomodulatory functions of PvTAMs.

There are two members of the HO family of enzymes expressed in humans and mice; HO-1 which is induced in response to stress stimuli, and HO-2 which is a non-inducible isoform (27). Enzymatically active HO-1 is associated with the smooth endoplasmic reticulum and breaks down heme to the biologically active catabolites biliverdin, ferrous iron (Fe^2+^) and carbon monoxide (CO)(27). HO-1 is expressed in a variety of cancers and has been associated with pro-metastatic (3), cytoprotective (28–30) and immune modulatory functions (9, 31, 32). However, to date, there have been no clinical trials targeting HO-1 or PvTAMs in cancer.

The only HO inhibitor that has been investigated clinically is tin mesoporphyrin (SnMP). SnMP has completed a Phase III trial for the treatment of neonatal hyperbilirubinemia (NCT02685137), used as a single dosing regimen to target hepatic HO activity, aiming to control excessive serum bilirubin levels (33). SnMP has a short serum half-life and is not orally bioavailable (34), which makes chronic daily dosing for cancer unfeasible. These hurdles highlight the need for novel HO inhibitors which are orally bioavailable for testing as a cancer immunotherapy. This preclinical study characterizes a next generation HO inhibitor, KCL-HO-1i that is an orally bioavailable small molecule immunotherapeutic for targeting HO-1 activity and PvTAM function in cancer.

## Results

### LYVE-1^+^ PvTAMs modulate the immune-landscape of cancer using HO-1 activity

**Figure 1:**
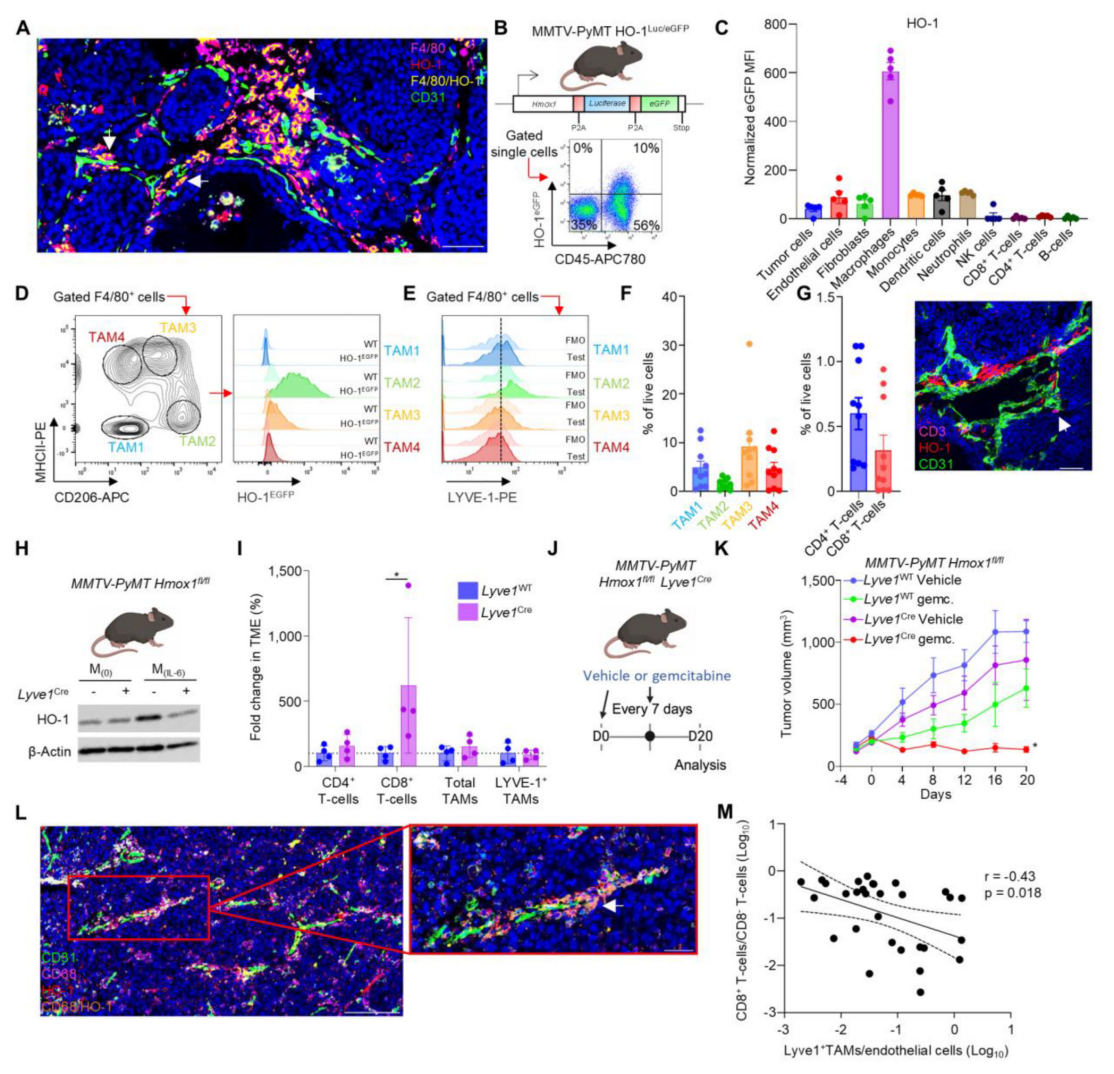
LYVE-1^+^ PvTAMs modulate the immune landscape and resistance to chemotherapy in cancer through their expression of HO-1. **(A)** Representative image of a frozen section of *MMTV-PyMT* tumor stained with DAPI (nuclei; blue) and antibodies against F4/80 (magenta), HO-1 (red), CD31 (green). Colocalizing pixels for F4/80 and HO-1 are shown in yellow. Scale bar is 50 µm. **(B-E)** Schematic depicting the transgene of the HO-1 reporter mice (HO-1^Luc/eGFP^) (top panel) and representative dot plot of FACs-gated live (7AAD^-^) cells from enzyme-dispersed *MMTV-PyMT* HO-1^Luc/eGFP^ tumors separated based on their respective expression of HO-1 (eGFP) and CD45 (bottom panel) **(B)**. HO-1/eGFP normalized median fluorescent intensity (MFI) of the indicated tumoral cell populations with background MFI subtracted from WT mice (n=5) **(C)**. Representative contour plot of FACs-gated live (7AAD^-^), CD45^+^F4/80^+^ TAMs separated based on their expression of CD206 and MHCII (left) and histograms showing the HO-1/eGFP expression of the subsets alongside the background fluorescence in WT mice (right) **(D)**. Representative histograms of the TAM populations identified in **(D)** for their surface expression of LYVE-1 alongside their respective fluorescence minus one (FMO) staining **(E). (F)** Quantitation of the respective TAM populations identified in **(D)** across *MMTV-PyMT* tumors (n=10). **(G)** Quantitation of T-cell populations (left) in FACs-gated live (7AAD^-^) cells from enzyme-dispersed *MMTV-PyMT* tumors (n=10) and representative image of a frozen section of *MMTV-PyMT* tumor (right) stained with DAPI (nuclei;blue) and antibodies against CD3 (magenta), HO-1 (red) and CD31 (green). Scale bar is 50 µm. **(H)** Western blot for HO-1 and β-actin expression in bone marrow derived macrophages (BMDMs) under M_(0)_ (CSF-1 alone) and IL-6 polarization (M_(IL-6)_) conditions from *MMTV-PyMT* x *Hmox1*^fl/fl^ mice with (*Lyve1*^Cre^) or without (*Lyve1*^WT^) cre-recombinase driven from the *Lyve1* promoter. **(I)** Tumors from *Lyve1*^WT^ and *Lyve1*^Cre^ *MMTV-PyMT Hmox1*^fl/fl^ mice were enzyme-dispersed and assessed using flow cytometry for the abundance of live (7AAD^-^) CD8^+^ and CD4^+^ T-cells (n=4 per group) relative to the *Lyve1*^WT^ animals. **(J-K)** Schematic representing the dosing strategy for gemcitabine in *Lyve1*^WT^ and *Lyve1*^Cre^ *MMTV-PyMT Hmox1*^fl/fl^ **(J)**. Growth curves of established spontaneous tumors that were given vehicle or gemcitabine (6.6mg/kg/7days) where indicated. Indicated dosing started at day zero (cohorts of n=5-8 mice) **(K). (L)** Representative image of a frozen section of human invasive ductal carcinoma stained with SYTO (nuclei; blue) and antibodies against CD68 (magenta), HO-1 (red), and CD31 (green). Colocalizing pixels for CD68 and HO-1 are displayed in orange. Scale bars represent 200 μm (left panel) and 50 μm (right panel). **(M)** Correlation of the association between LYVE-1^+^ PvTAMs and ratio CD8^+^:CD8^-^ T-cell infiltration from ScRNAseq data (described in methods). Images in panels **(B), (H)** and **(J)** were created using *BioRender* software. Bar charts represent the mean, error bars SD, and the dots show individual data points from individual tumors in mice or human patients as indicated. Gemc.; gemcitabine. * *P*<0.05

PvTAMs in the *MMTV-PyMT* spontaneous murine model of breast cancer express the heme-degrading enzyme HO-1 (Figure 1A and S1A). PvTAMs are characterized by their LYVE-1^+^CD206^hi^MHCII^lo^HO-1^hi^ phenotype in *MMTV-PyMT* tumors (Figure 1B-E and S1A-C)(9). LYVE-1^+^ TAMs account for 1.5 ± 1.0% total live tumoral cells (Figure 1F) and are associated with an immune ‘cold’ TME with only 0.6 ± 0.4% CD4^+^ and 0.3 ± 0.4% CD8^+^ T-cells (Figure 1G and S1D). T-cell infiltration is pivotal to the response of immune checkpoint blockade (35–37) and immune-stimulating chemotherapies (38, 39). Genetic inactivation of HO-1 in LYVE-1^+^ PvTAMs in *MMTV-PyMT* animals on a *Hmox1*^fl/fl^ background with a *Lyve1* promoter driven Cre-recombinase (referred to as *Lyve1*^Cre^ or *Lyve1*^WT^ mice, respectively) resulted in a specific infiltration of CD8^+^ T-cells (621 ± 520%) into the TME (Figure 1H-I and S1E), highlighting a pivotal role for HO-1 activity in PvTAMs in modulating the immunological ‘heat’ of the TME. There is a growing appreciation of the role of standard-of-care chemotherapies in both priming T-cell responses and improving T-cell migration into the TME (40–45). Administering gemcitabine (as a clinically relevant single-agent chemotherapy), at an allometrically scaled clinically relevant dose and regimen to *Lyve1*^Cre^ mice (Figure 1J) delivered long-term durable control of tumor growth compared to *Lyve1*^WT^ mice that were insensitive to gemcitabine treatment (Figure 1K). HO-1 expressing PvTAMs in human breast cancer (Figure 1L) and their prevalence across the endothelium correlates inversely with the ratio of CD8^+^:CD8^-^ T-cells within the TME (Figure 1M). These observations highlight HO-1 as a therapeutic target associated with PvTAM function in the TME.

### KCL-HO-1i is an orally bioavailable inhibitor of HO-1

**Figure 2:**
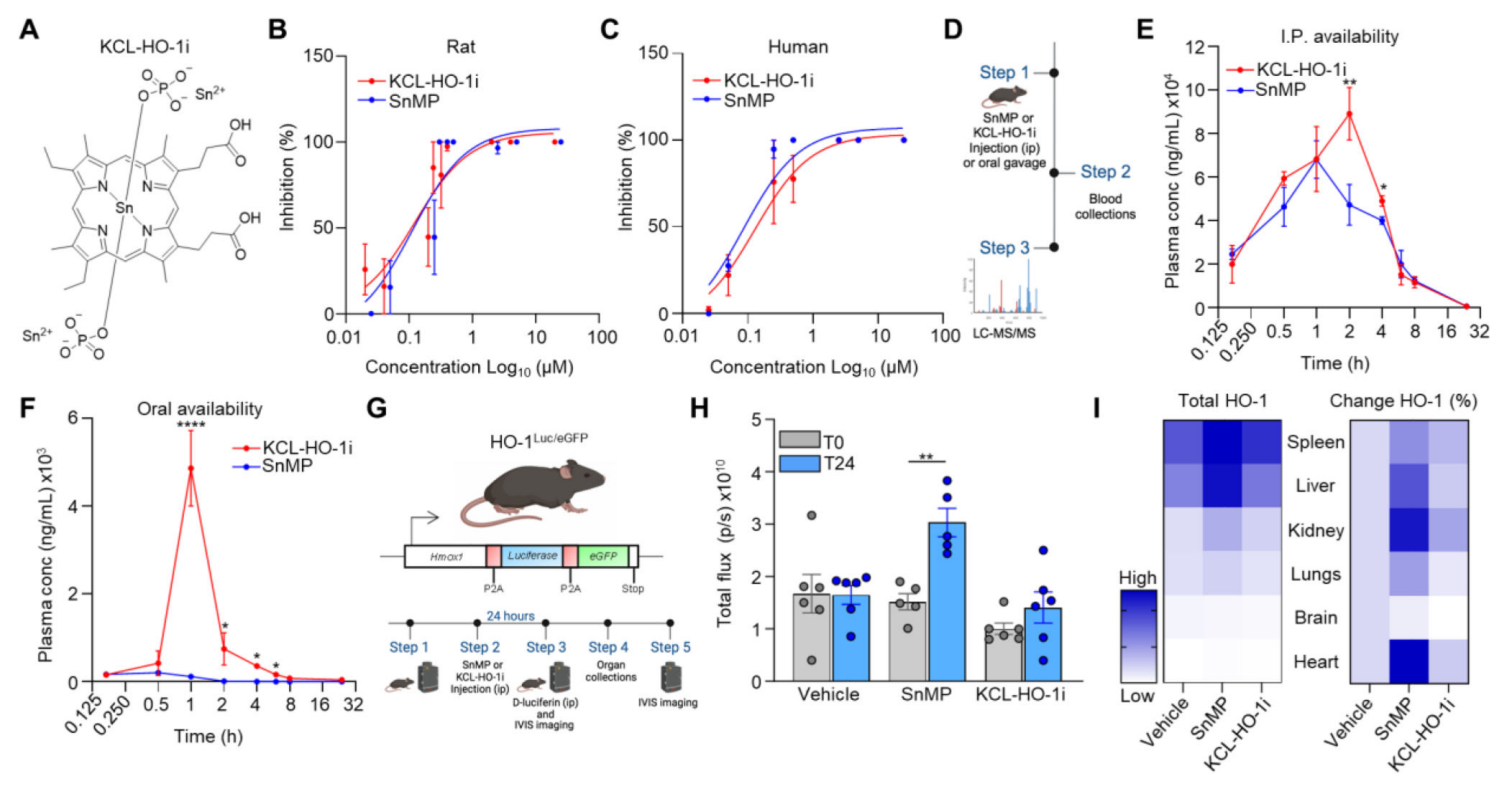
KCL-HO-1i is an orally bioavailable HO-1 inhibitor. **(A)** Chemical structure of KCL-HO-1i. **(B-C)** *In vitro* HO-1 activity in response to a dose escalation of KCL-HO-1i or SnMP using either rat splenic microsomes (**B**) or human hemin-induced HO-1 expressing HEK293T cell lysates **(C)** (n=3 biological repeats). **(D-F)** Schematic representing the dosing strategy and experimental protocol for the PK analysis **(D)** and plasma concentrations of KCL-HO-1i and SnMP at the indicated times post-delivery of 25µMol/kg via i.p **(E)** or gavage feeding **(F)** routes of administration (n=3 mice at each time point). **(G-I)** Schematic depicting the transgene of the HO-1 reporter mice (HO-1^Luc/eGFP^) (upper panel) and the experimental outline (below) **(G)**. Luciferase expression was measured in HO-1^Luc/eGFP^ reporter mice at time 0 (baseline) and 24 h post administration with either KCL-HO-1i (25µMol/kg), SnMP (25µMol/kg) or vehicle control. Whole mouse total photon flux at each timepoint and condition was quantitated **(H)**. Heat map displaying luciferase expression across the indicated dissected tissues (left panel) and their percentage change relative to vehicle treated animals (right panel) **(I)** (cohorts of n=6 mice). Images in panel **(D)** and **(G)** were created using *BioRender*. Bar charts show the mean, error bars SD, and the dots show individual data points from individual mice. Line charts display the mean and SEM. * *P*<0.05, ** *P*<0.01, **** *P*<0.0001.

Metalloporphyrin molecules have been used effectively as preclinical drugs for targeting HO-1 activity (46). Although many modifications have been made to the porphyrin backbone, we explored the impact of switching the tin chloride salt of SnMP to a tin phosphate salt formulation to improve the solubility of the molecule (Figure 2A and S2), which we refer to here as KCL-HO-1i. The phosphate salts are expected to have a better solubility profile than the chloride salt due to their higher hydration energy, attributed to the larger size and higher charge of the phosphate ion compared to the chloride ion. In an *in vitro* HO-1 activity assay, SnMP and KCL-HO-1i had a similar IC_50_ of 111 nM and 123 nM against rat HO-1 respectively (Figure 2B) and 84 nM and 128 nM against human HO-1 respectively (Figure 2C). Furthermore, KCL-HO-1i displayed good selectivity and minimal off-target activity in a safety screen of 44 receptors and enzymes with only some inhibitory (61% inhibition) activity to phosphodiesterase 3A at a high (10 μM) concentration of the compound (Table S1). Comparing the pharmacokinetic (PK) characteristics of KCL-HO-1i and SnMP via intraperitoneal (i.p.) delivery to mice *in vivo* demonstrated that KCL-HO-1i had a slower time to maximum serum concentration (T_max_; 2 h vs. 1 h respectively), higher maximum serum concentration (C_max_; 89 ± 27 µg/mL vs. 68 ± 19 µg/mL respectively) and comparable serum half-life (T_1/2_; 3.9 vs. 4 h respectively) (Figure 2D-E). When delivered via oral administration (per os; p.o.), KCL-HO-1i was found to be bioavailable with a T_max_ at 1 h, C_max_ of 4.9 ± 1.5 µg/mL and a T_1/2_ of 6.3 h, whereas SnMP, as anticipated, was not orally bioavailable (Figure 2D and 2F). At an equivalent dose of KCL-HO-1i the C_max_ of oral delivery was approximately 18-fold lower than the dose achieved through i.p. delivery. Some metalloporphyrin compounds can induce the expression of HO-1 (27). This is an undesirable attribute for a HO-1 inhibitor as the newly expressed enzyme is biological active. To investigate whether KCL-HO-1i induced HO-1 expression we injected KCL-HO-1i into non-tumor bearing HO-1^Luc/eGFP^ reporter mice and measured luciferase (Luc) expression using bioluminescence analysis (as a surrogate of HO-1 expression) over a 24 h period (Figure 2G). At a whole-body level KCL-HO-1i treated animals displayed a stable HO-1 expression after 24 h of exposure to the compound (Figure 2H). At the tissue-level, the major HO-1 expressing organs were the spleen > liver > kidney at baseline (Figure 2I). However, KCL-HO-1i did not significantly increase the expression of HO-1 in any of these tissues analyzed (Figure 2I). These data highlight that KCL-HO-1i is an orally bioavailable inhibitor for HO-1 with favorable PK and biological parameters.

### KCL-HO-1i potentiates the immune-stimulatory properties of cytotoxic chemotherapy

**Figure 3:**
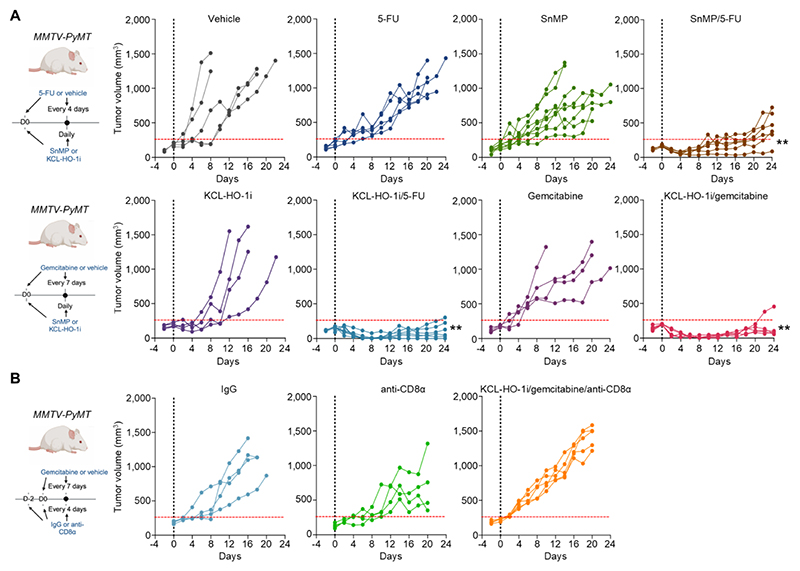
KCL-HO-1i synergizes with chemotherapy to achieve immunological control of tumor growth. (**A**) Schematic representing the i.p. dosing strategy for KCL-HO-1i (25 µMol/kg/day) or SnMP (25 µMol/kg/day) and/or 5-FU (40 mg/kg/4 days) or gemcitabine (6.6 mg/kg/7 days) and/or vehicle in *MMTV-PyMT* mice bearing established tumors (left) and the individual tumor growth curves for the respective treatment (right). (**B**) Schematic representing the i.p. dosing strategy for non-immune IgG and immune-depleting anti-CD8α antibodies that were also given alongside KCL-HO-1i (25 µMol/kg/day) and gemcitabine (6.6 mg/kg/7 days) and in *MMTV-PyMT* mice. Vertical dashed black lines mark the start of treatment (day 0) and horizontal red line marks 250 mm^3^. Solid lines represent individual tumors and mice. Images in panel **(A)** and **(B)** were created using *BioRender*. ** *P*<0.01.

To evaluate KCL-HO-1i as an anti-cancer therapeutic for targeting PvTAMs in cancer we treated *MMTV-PyMT* mice bearing established tumors with KCL-HO-1i using i.p. delivery (to allow a side-by-side comparison with SnMP). As expected, HO-1 inhibition using either KCL-HO-1i or SnMP did not control tumor growth as single agents (Figure 3A). We initially paired KCL-HO-1i with 5-fluorouracil (5-FU), a chemotherapy we have previously demonstrated to synergize with SnMP (31). KCL-HO-1i and 5-FU combined synergistically, delivering durable control of tumor growth in *MMTV-PyMT* mice (Figure 3A). All mice responded equivalently despite the spontaneous nature of the model. This response was not chemotherapy class-specific as KCL-HO-1i given alongside gemcitabine also delivered a durable control of tumor growth (Figure 3A), which paralleled the observation using genetic inactivation of HO-1 in *MMTV-PyMT* mice (Figure 1K), suggesting PvTAMs were the therapeutic target of KCL-HO-1i. To investigate the synergy between KCL-HO-1i and chemotherapy in a second model, we utilized subcutaneous MN-MCA1 sarcoma tumors (47), which contain HO-1 expressing LYVE-1^+^CD206^hi^ TAMs (Figure S3A-C) that reside in the Pv niche (Figure S3C). In this model, KCL-HO-1i also synergized with gemcitabine to deliver a durable control of tumor growth (Figure S3D).

To evaluate the immune-dependency of the observed tumor control between KCL-HO-1i and gemcitabine, *MMTV-PyMT* mice bearing established tumors were depleted of CD8^+^ T-cells prior to initiating KCL-HO-1i/gemcitabine treatment. In the absence of CD8^+^ T-cells, KCL-HO-1i/gemcitabine failed to control tumor growth (Figure 3B and Figure S3E-F), highlighting the fact that tumor control elicited by this dual therapy combination was immune-dependent. As such, KCL-HO-1i in combination with chemotherapy provides an immunotherapeutic drug combination.

### KCL-HO-1i and chemotherapy act synergistically to improve CD8^+^ T-cell responses against cancer

**Figure 4:**
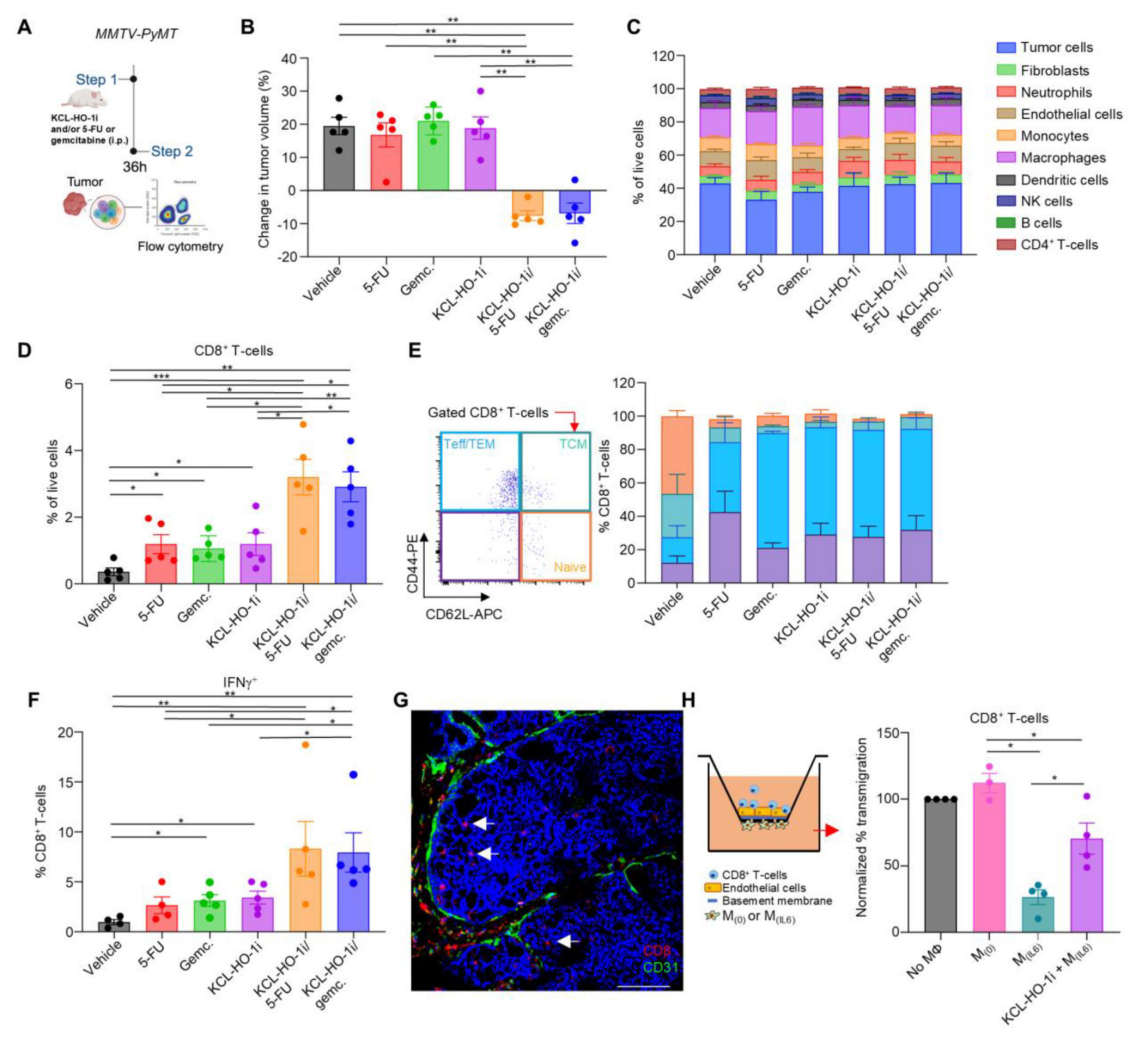
KCL-HO-1i and chemotherapy create an immunologically hot TME. KCL-HO-1i and/or 5-FU or gemcitabine and/or vehicle were administered to *MMTV-PyMT* mice bearing 500-700 mm^3^ tumors. At 36 h post treatment initiation of treatment mice were sacrificed and tumors were harvested and analyzed. **(A)** Schematic representing the dosing strategy. **(B)** The fold change of tumor growth over 36 h post initiation of treatment. (**C-G**) Tumors were enzyme-digested to release single cells which were analyzed for their live (7AAD^-^) stromal cell composition using flow cytometry (n=5 tumors per group) (**C**) and CD8^+^ T-cells **(D)**. Gating strategy (left panel) and quantitation of CD8^+^ T-cell subsets based on CD62L and CD44 expressions (right panel; TCM; T central memory cells, TEM; T effector memory cells, Teff; T effectors cells) **(E)**, and the ability of the tumoral CD8^+^ T-cells to secrete IFNγ post *ex vivo* exposure to PMA/ionomycin treatment **(F). (G)** Representative image of a frozen section of *MMTV-PyMT* tumor treated with KCL-HO-1i and gemcitabine stained with DAPI (nuclei;blue) and antibodies against CD8 (red) and CD31 (green). Scale bar is 100 µm and white arrows indicate infiltrating CD8^+^ T-cells. **(H)** Schematic of the perivascular niche assay (left) and the relative transendothelial migration of CD8^+^ T-cells in the presence or absence of M_(0)_ or M_(IL-6)_ cells on the basolateral surface with or without 25 µM KCL-HO-1i as indicated. Image in panel **(A)** was created using *BioRender*. Bar charts show the mean, error bars SD, and the dots show individual data points from individual tumors and mice. Gemc.; gemcitabine. * *P*<0.05, ** *P*<0.01, *** *P*<0.001.

We next investigated the TME of *MMTV-PyMT* tumors which had been treated for 36 h (Figure 4A) for characterizing the earliest biological changes in response to KCL-HO-1i. As expected after 36 h of treatment, tumor remission was only observed in mice that had received both KCL-HO-1i and 5-FU or gemcitabine (Figure 4A). Tumors were excised, and enzyme-digested to assess the cellular composition of the TME. The cellular component of the TME was remarkably similar post treatment (Figure 4C, S4 and S5A). However, KCL-HO-1i, 5-FU and gemcitabine as single-agent therapies resulted in an increase in the abundance of CD8^+^ T-cells in the TME (Figure 4D), which mirrored the biological observations using genetic inactivation of HO-1 in PvTAMs (Figure 1H-J). Analysis of tumor tissue sections from these mice revealed evidence of CD8^+^ T-cell infiltration into tumor regions with no evidence of retention in the Pv niche when HO-1 was either inhibited with KCL-HO-1i or genetically inactivated (Figure S5B). Quantitation of these tumor sections for CD8+ T-cells also mirrored the results of the flow cytometry analysis (Figure 4D and S5C). When KCL-HO-1i was combined with either 5-FU or gemcitabine there was a synergistic enhancement in the prevalence of CD8^+^ T-cells in the TME (Figure 4D and S5C), which was associated with tumor control (Figure 4B). Analysis of the CD8^+^ T-cell subsets revealed a treatment related reduction in the number of naïve CD44^-^CD62L^+^ T-cells and increase in the number of CD44^+^CD62L^-^ T-effector and -effector memory cells in the CD8^+^ compartment (Figure 4E). KCL-HO-1i also resulted in an increased proportion of CD8^+^ T-cells capable of expressing IFNγ within the TME, an effect that was further boosted when KCL-HO-1i was administered alongside 5-FU or gemcitabine (Figure 4F), where CD8^+^ T-cells could be found infiltrating the tumor (Figure 4G). To investigate this infiltration in a second model we analyzed the TME of subcutaneous MN-MCA1 sarcoma tumors which had been treated for 36 h (Figure S5D). Consistent with observations in the *MMTV-PyMT* tumors, gemcitabine and KCL-HO-1i synergized to improve CD8^+^ T-cell infiltration to tumors (Figure S5E). Analysis of a panel of activation and exhaustion markers on these T-cells revealed relatively few changes that could be ascribed to the dual therapy other than a significant reduction in CD8^+^ T-cells expressing the T cell immunoglobulin and mucin domain-containing protein 3 (TIM3) (Figure S5F), which is an inhibitory immune checkpoint receptor that marks the most dysfunctional CD8^+^ T-cells (48). In light of the high number of PD-1 expressing T-cells in this model (∼60%) (Figure S5F) we investigated whether KCL-HO-1i could synergize with an immune checkpoint blockade of PD-1 receptors (49) in MN-MCA1, however, no evidence of synergy was observed (Figure S5F-G), suggesting a discrete mechanism of action when combined with chemotherapy in this model. We found no evidence *in vitro* that KCL-HO-1i, heme or the catabolites of heme degradation can directly modulate IFNγ production by CD8^+^ T-cells (Figure 4G). As such, the increase CD8^+^ T-cells capable of expressing IFNγ may reflect the newly recruited nature of these cells to the TME.

To gain further mechanistic insight into the association of KCL-HO-1i in modulating PvTAM function to facilitate the CD8+ T-cells entry to the TME, we investigated an in vitro Pv niche assay we previously published (9). In this assay IL-6 exposed bone marrow derived macrophages, which are analogous to the LYVE-1^+^ HO-1^+^TAMs were seeded onto the basolateral side of a transwell insert, and then a basement membrane and an endothelial layer were seeded on the apical side of the insert (Figure 4H). CD8^+^ T-cells were then seeded onto the endothelial cell layer and their transmigration quantified. As previously identified M_IL6_, but not M_0_, macrophages restrict CD8^+^T-cells from transmigrating across the endothelial layer (Figure 4H), which we previously demonstrated to be HO-1-dependent (9). In agreement with this, when KCL-HO-1i was added, CD8^+^ T-cell migration was improved (Figure 4H), highlighting KCL-HO-1i as a therapeutic for modulating PvTAM function in immune exclusion.

### KCL-HO-1i rewires the TME to support tumor control

**Figure 5:**
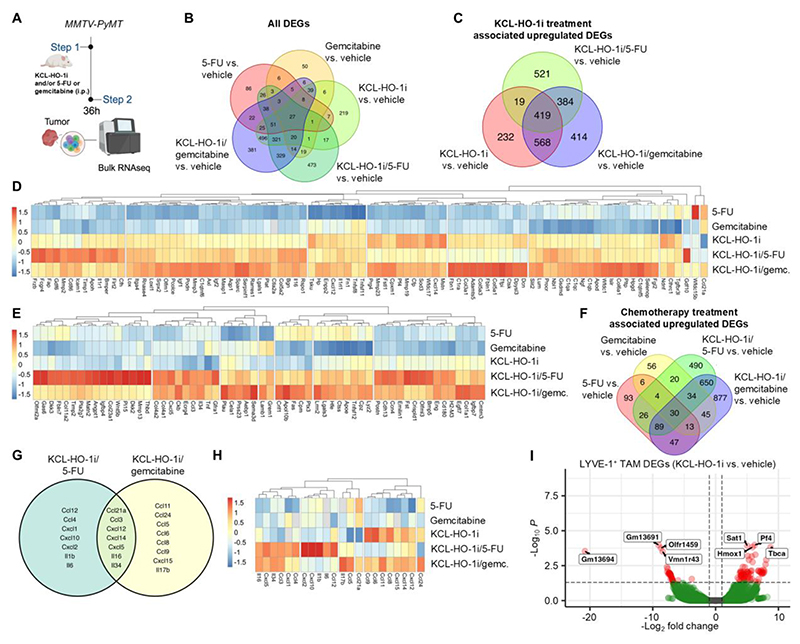
KCL-HO-1i rewires the TME of breast cancer. **(A)** Schematic representing the i.p. dosing strategy for mice bearing established *MMTV-PyMT* tumors which were treated with KCL-HO-1i (25µMol/kg/day) and/or 5-FU (40mg/kg) or gemcitabine (6.6mg/kg) and/or vehicle. Tumor tissue (cohorts of n=5 mice and tumors),) was analyzed at 36 h post treatment initiation by bulk RNAseq. **(B)** Venn diagram showing all upregulated DEGs in the tumor TME for the respective treatments against vehicle-treated mice and their intercepts between groups. **(C-E)** Venn diagram showing all upregulated tumor tissue DEGs for KCL-HO-1i treated mice against vehicle-treated animals and their intercepts between groups **(C)**, heatmap of hierarchical clustered common upregulated DEGs between treatment groups (419 genes) that are secreted genes (91 genes (Figure S7A)) across treatments **(D)**, and heatmap of hierarchical clustered common upregulated DEGs for KCL-HO-1i/chemotherapy (384 genes) that are secreted genes (60 genes (Figure S7D)) **(E). (F-H)** Venn diagrams showing all upregulated DEGs for chemotherapy treated animals **(F)** and chemokine and upregulated DEGs associated dual therapy treated animals **(G)** against vehicle-treated animals and their intercepts between groups. (**H**) Heatmap of hierarchical clustered chemokine and cytokine upregulated DEGs associated with KCL-HO-1i treatment. **(I)** Volcano plot showing DEGs from bulk RNAseq of FACs-sorted LYVE-1^+^ TAM from *MMTV-PyMT* tumors treated for 36 h with Vehicle or KCL-HO-1i (25µMol/kg/day) (cohort of n=3 mice; red dots show significantly regulated gene changes). Image in panel **(A)** was created using *BioRender*. Gemc.; gemcitabine.

In light of the increase in effector-ready IFNγ expressing T-cells entering the TME with KCL-HO-1i and chemotherapy (Figure 4D), we sought to explore which catabolite of heme degradation might account for this observation. *In vitro* we exposed CD8^+^ T-cells to KCL-HO-1i, hemin and the heme degradation catabolites CO, biliverdin and Fe^2+^, but found no evidence of a direct role for any of these conditions modulating IFNγ production (Figure S5I). As such, we next explored the transcriptional profiles of the TME using bulk RNAseq analysis (Figure 5A). Analysis of the differentially expressed genes (DEGs) in the TMEs highlighted a divergent gene expression pattern associated with the individual treatments and combination approaches (Figure 5B). A total of 419 upregulated DEGs were specifically associated with KCL-HO-1i treatment (Figure 5C), of which 92 were secreted molecules (Figure 5D and S6A). These upregulated DEGs could represent potential pharmacodynamic gene signatures for KCL-HO-1i targeting HO-1 activity within the TME. Gene ontology analysis revealed a diverse range of pathways associated with the response to KCL-HO-1i. Interestingly, there are several pathways associated with blood vasculature, such as blood vessel angiogenesis and morphogenesis (Figure S6B-C), linked to the pro-angiogenic function of PvTAMs (18) and a role of HO-1 in this process (27). A total of 384 upregulated DEGs were specifically associated with the combination of KCL-HO-1i with 5-FU or gemcitabine (Figure 5C), of which 60 were secreted molecules (Figure 5E and S6D), that were also heavily associated with vasculature-related processes (Figure S6E). In contrast, 5-FU and gemcitabine had a minor impact on the TME as single agents with only 30 DEGs associated with the response to chemotherapy across the treatment groups (Figure 5F), of which only 3 were secreted proteins (Figure S6F). Considering that the largest cellular change in the TME associated with KCL-HO-1i treatment with 5-FU and gemcitabine was a significant increase in the infiltration of CD8^+^ T-cells (Figure 4D), we explored the chemokines that were associated with both dual treatments (Figure 5G-H). Of these genes, C*cl21a*/Ccl21 (50), *Cxcl12/*Cxcl12 (at low doses) (51) and *Cxcl14*/Cxcl14 (52) have been associated with T-cell migration to the TME and could represent interesting molecules to account for the CD8^+^ T-cell infiltration observed in KCL-HO-1i/chemotherapy treated tumors.

HO-1 inhibition in TAMs has been observed to result in their expression of a more proinflammatory phenotype (53). Therefore, we investigated whether KCL-HO-1i could modulate the phenotype of the PvTAM subset. We sorted LYVE-1^+^ TAMs from *MMTV-PyMT* tumors from mice treated with KCL-HO-1i or vehicle for 36 h and then performed bulk RNAseq on this population (Figure S7A). Consistent with the lack of change in the abundance of the PvTAM subset with KCL-HO-1i treatment (Figure S6B), the phenotype of the population remained transcriptionally stable (Figure 5I and S7), and retained a transcriptional profile similar to the untreated LYVE-1^+^ TAM population (Figure S7B-D). Notably, *Hmox1* gene expression was a differentially expressed gene, although KCL-HO-1i does not increase HO-1 protein translation (Figure 2H-I), the observed increase in gene expression, however, confirms that KCL-HO-1i was effectively targeting these cells.

### KCL-HO-1i is an orally bioavailable immunotherapeutic with favorable toxicity profile

**Figure 6:**
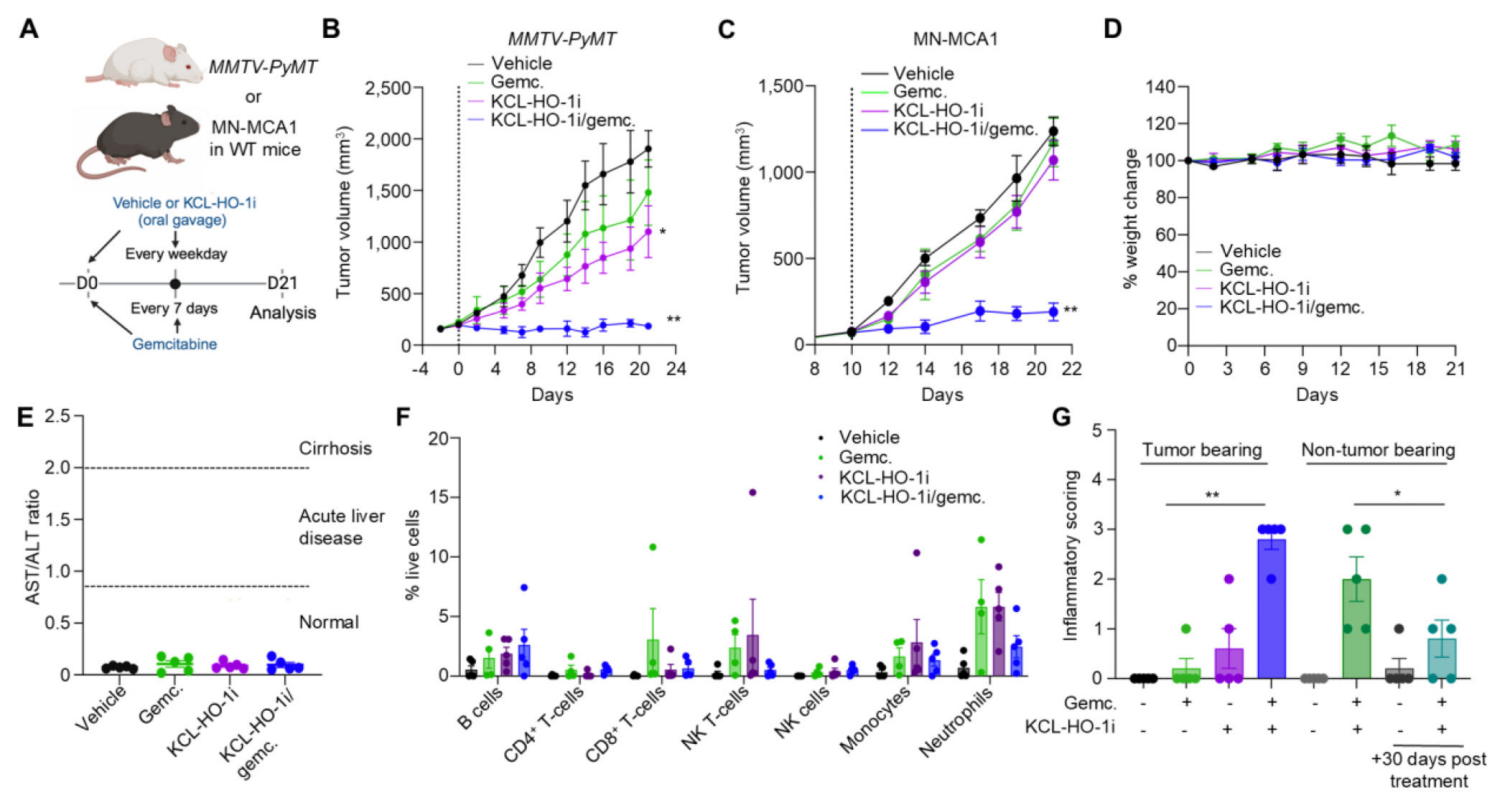
Orally delivered KCL-HO-1i is non-toxic and synergizes with gemcitabine to control tumor growth. **(A-C)** Schematic representing the p.o. dosing strategy for KCL-HO-1i (25µMol/kg/day) and/or the i.p. dosing for gemcitabine (6.6 mg/kg/7 days) and/or vehicle in *MMTV-PyMT* mice bearing established spontaneous tumors or C57Bl/6 mice bearing ectopic MN-MCA1 tumors (cohorts of n=5-6 mice) **(A)** and tumor growth curves (vertical black dashed line represents treatment initiation) for *MMTV-PyMT* **(B)** and *MN-MCA1* **(C)** respectively. **(D-G)** The *MMTV-PyMT* mice in **(B)** were evaluated for evidence of toxicity, including weight change from treatment initiation **(D)**, end of treatment (day 21) plasma ratio of AST:ALT enzymes **(E)**, blood circulating immune cells assessed using flow cytometry **(F)** and pathology assessment of inflammatory cell infiltrate in the lungs alongside an additional cohort or WT non-tumor bearing mice given dual treatment for 21 days and then assessed at treatment cessation or 30 days after treatment cessation **(G)**. Image in panel **(A)** was created using *BioRender*. Bar charts show the mean, error bars SD, and the dots show individual data points from individual tumors and mice. Line charts display the mean and SEM. Gemc.; gemcitabine. * *P*<0.05, ** *P*<0.01.

Having established KCL-HO-1i as a small molecule that can target the immunomodulatory function of the PvTAM population we explored KCL-HO-1i as an orally bioavailable immunotherapeutic that would be feasible for daily delivery as an anti-cancer therapeutic. We treated tumor bearing *MMTV-PyMT* mice with KCL-HO-1i using p.o. delivery alongside gemcitabine (Figure 6A). Despite relatively lower oral bioavailability compared to i.p. route (Figure 2D-F), p.o. delivery of KCL-HO-1i combined with gemcitabine delivered durable control of tumor growth in *MMTV-PyMT* mice (Figure 6B). Unexpectedly, KCL-HO-1i also slowed tumor growth as a single agent in this model (Figure 6B). Using the subcutaneous ectopic MN-MCA1 sarcoma model, p.o. delivery of KCL-HO-1i and i.p. delivery of gemcitabine in mice bearing established MN-MCA1 tumors also combined synergistically to deliver tumor control in this model (Figure 6C). P.o. delivery of KCL-HO-1i did not result in any obvious signs of ill health in the animals, which was assessed through their maintenance of weight (Figure 6D), lack of adverse clinical signs (Figure S8A) and plasma abundance/ratio of the liver enzymes alanine aminotransferase (ALT) and aspartate aminotransferase (AST) (as a surrogate of liver toxicity) which were similar to controls (54) (Figure 6E and S6B-C). Equally, the immune cell composition of the blood was unaffected by any of the treatment conditions, suggesting there was no observable toxicity of KCL-HO-1i and/or gemcitabine on the bone marrow (Figure 6F). After 21 days of treatment, the brain, heart, kidney, liver, and lung tissues from mice were harvested and formalin-fixed and paraffin-embedded. H&E-stained sections from these tissues were then assessed by a trained pathologist. Both single KCL-HO-1i and KCL-HO-1i/gemcitabine treated groups had no observable effects on the brain, heart, liver, and kidneys (Table S2). However, the lungs of KCL-HO-1i/gemcitabine dual-treated animals did display evidence of mild inflammation as noted by immune infiltrates (largely mononuclear cells) in the Pv region (grade 2-3) (Figure 6G, S6D and Table S2). HO-1 is expressed in the lungs within tissue resident F4/80^+^ macrophages (9) and as such, potentially these observations highlight a protective role of these cells in the context of exposure to cytotoxic agents. Mice in the KCL-HO-1i/gemcitabine treated group had no observable lung metastasis, which may be reflective of the small size of the tumors at the end of treatment in this cohort. To investigate whether the inflammation observed in the lung was a reversible effect, we gave KCL-HO-1i/gemcitabine to non-tumor bearing mice and analyzed lung tissue at the end of treatment and 30-days after the cessation of treatment (Figure 6G). There were clear signs of the inflammation resolving in the lungs after the cessation of treatment, highlighting that the observed inflammation was indeed reversible and did not leave any evidence of fibrosis (Figure 6G and Table S3).

These data demonstrate that KCL-HO-1i combines synergistically with gemcitabine to deliver robust and durable control of tumor growth with limited toxicity. As such, KCL-HO-1i represents a translatable therapeutic for targeting the immune suppressive activity of PvTAMs in cancer.

## Discussion

PvTAMs facilitate a variety of pro-tumoral processes (20, 21) and their relative resistance to CSF1-CSF1R blockade (25, 26) highlights a need for therapeutic approaches which selectively target this myeloid population within the TME. HO-1 has emerged as a therapeutic target for the immunomodulatory function of LYVE-1^+^ PvTAMs (9, 31). However, there are no orally bioavailable HO inhibitors in clinical development or use. Unlike more traditional immune checkpoint immunotherapies which are IgG-based biologics (such as those which target CTLA-4 and PD-1/PD-L1) and have serum half-lives measured in weeks (55), small molecules (such as the metalloporphyrins) are rapidly metabolized and cleared with half-lives in hours. As such, it would not be feasible to administer small molecule immunotherapeutic to a patient through parenteral routes on a daily basis in a clinical setting. We demonstrate that KCL-HO-1i successfully overcomes the bioavailability challenges of current HO-1 inhibitors and achieves a therapeutic dose as an anti-cancer agent through oral delivery. Interestingly, the maximum serum concentration of KCL-HO-1i achieved through oral delivery was 18-fold lower than with intraperitoneal (i.p.) delivery but still delivered equivalent tumor control, suggesting that the therapeutic concentration of KCL-HO-1i may be lower than anticipated. This effect is likely facilitated by the absence of HO-1 induction in response to inhibition by KCL-HO-1i, a phenomenon observed with SnMP. Furthermore, the good selectivity and minimal off-target interactions observed for KCL-HO-1i are encouraging from a therapeutic development standpoint. Thus, KCL-HO-1i addresses key challenges faced by current HO-1 inhibitors, making it a promising candidate for targeting the pro-tumoral functions of PvTAM in cancer.

LYVE-1^+^ PvTAMs cells form CCR5-dependent multicellular ‘nests’ that support their biological function in a collaborative biological unit (9). As such, these cells act in a ‘gatekeeper’ role excluding T-cells from the TME that is facilitated through their spatial location at the blood-tumor interface. We demonstrate that KCL-HO-1i, through targeting the activity of PvTAMs, can both elicit T-cell infiltration into the TME and increase the abundance of IFNγ-expressing T-cells with effector function. The immune ‘heat’ of a TME has been linked to better prognosis (56, 57) and response following both adjuvant chemotherapy (56, 58) and immune checkpoint blockade (35, 59). The biological changes in the TME in response to KCL-HO-1i mirror those observed through genetic inactivation of HO-1 in *Hmox1*^fl/fl^ mice with *Lyve1*^Cre^ in the *MMTV-PyMT* tumor model, affirming PvTAMs as a key biological target of KCL-HO-1i within the TME.

We show that KCL-HO-1i synergizes with gemcitabine to deliver durable control of tumor growth in both spontaneous *MMTV-PyMT* breast tumors and an ectopic MN-MCA1 sarcoma model. Gemcitabine is a deoxycytidine analogue that prevents DNA synthesis and is used to treat a range of tumors including bladder, breast, esophageal, non-small cell lung, ovarian, pancreatic, and testicular cancers. Gemcitabine has also been shown to play a variety of immune modulatory effects in cancer (44), such as depleting Tregs (60), improving CD8^+^/CD4^+^ T-cell infiltration (61), depleting immune suppressive myeloid populations (62) and improving tumor cell sensitization to immune recognition (63, 64). KCL-HO-1i in combination with gemcitabine represents an immunotherapy combination as both T-cell infiltration and effector function are boosted in the context of the dual therapy and the tumor control is dependent on CD8^+^ T-cells.

Interestingly, while HO-1 inhibition exerts anti-tumor effects, carbon monoxide (CO), a product of HO-1 activity, has also been reported to exhibit anti-tumor properties (65, 66) and to stimulate CD8^+^ T-cell responses (67). However, we did not find a direct link between KCL-HO-1i, heme or the HO-1-derived heme catabolites to account for the increased IFNγ production by the T-cells that we observed in the TME. Instead, our data reveal a broader role of KCL-HO-1i in re-wiring the TME *in vivo* which could underlie the enhanced anti-cancer immune response and improved immune landscape. When analyzing the transcriptional profile of the TME post treatment it was apparent that most of the transcriptional changes observed in the TME in the acute phase of tumor regression (36 h post initiation of treatment) were driven by KCL-HO-1i (419 upregulated DEGs) compared to chemotherapy (common to both gemcitabine and 5-FU; 30 common upregulated DEGs) across treatment cohorts. However, specific biological responses were also unique to the dual therapy (384 upregulated DEGs) which supports the concept that KCL-HO-1i and gemcitabine work in synergy, as opposed to just an additive combination effect.

KCL-HO-1i in combination with gemcitabine was well tolerated. HO-1^+^ F4/80^hi^ tissue-resident macrophages reside in a variety of organs and tissues (9). In these tissues, F4/80^hi^ macrophages were shown to be the only cells expressing HO-1 in the lungs (9), therefore, these cells are likely responsible for the homeostatic functions ascribed to HO-1 in healthy tissues (27). HO-1 is generally considered as a stress- or inflammation-inducible enzyme (30, 68) and its constitutive expression in tissues during homeostatic conditions is intriguing. However, KCL-HO-1i and gemcitabine did not result in any clinical toxicity in the animals after 21 days of treatment as a single agent. However, there was mild inflammation in the lungs at the end of treatment when KCL-HO-1i was combined with gemcitabine. Notably, gemcitabine has been demonstrated to cause pulmonary inflammation in approximately 23% of patients (69, 70), and this might suggest that HO-1 activity in the lungs may play a role in suppressing this inflammation as it was only observed when HO-1 was inhibited. Importantly, the observed inflammation was mild and did not result in any apparent signs of ill-health in the animals.

In summary, we show that LYVE-1^+^ TAMs, and their expression of HO-1, represent an immunotherapeutic target in cancer progression. We present KCL-HO-1i, a small-molecule therapeutic that overcomes the limitations of current HO-1 inhibitors for oncology indications and offers an orally bioavailable, non-toxic small-molecule drug candidate to target the pro-tumorigenic function of PvTAMs in cancer, specifically to alleviate resistance to standard-of-care chemotherapy.

## Materials and Methods

### Animals

Mice used in this study were housed under specific pathogen-free conditions in individually ventilated cages at a temperature of 21°C with water and food *ad libitum*. The transgenic (Tg) mice *Lyve-1* driven Cre recombinase (Cre) mice (B6;129P2-*Lyve1tm1.1(EGFP/cre)Cys* /J) were on a C57Bl/6 background and *MMTV-PyMT* mice were on a FVB/N background (71) and were obtained from The Jackson Laboratory. *Hmox1*^fl/fl^ mice were a gift from Professor George Kollias, Biomedical Sciences Research Center, Athens, Greece. The HO-1-Luciferace-eGFP reporter mice (HO-1^Luc/eGFP^) were on a C57Bl/6 background and generated in-house as described previously (9). The *Hmox1*^fl/fl^ (72) and *Lyve1-*Cre mice were crossed with *MMTV-PyMT* mice for the *MMTV-PyMT/Hmox1*^fl/fl^*Lyve1*^Cre^ (referred to as *Lyve1*^Cre^ mice in the manuscript). The HO-1^Luc/eGFP^ mice were crossed with *MMTV-PyMT* mice and F1 hybrid mice were used. Male ICR adult mice (∼21-25 g) were used for the PK analyses. Wild type female C57Bl/6 mice (21-25 g) for subcutaneous tumor experiments and male Sprague-Dawley rats (150-200 g weight) for microsome preparations were purchased from Charles River. Cohort sizes for experiments were informed by prior studies (9). Experiments were performed in at least duplicate (biological replicates) and for spontaneous *MMTV-PyMT* tumor studies, individual mice were collected on separate days and all data points were presented. *MMTV-PyMT* mice were approximately 24-28 g when tumors became palpable. End points for *MMTV-PyMT* tumor studies were assessed on a welfare assessment, however, were typically when the primary tumor either reached 2,000mm^3^ or at least 20 days on treatment (unless stated otherwise).

### Cell lines

MN-MCA1 sarcoma cells (47) were kindly gifted by Professor Antonio Sica, Humanitas Clinical and Research Center, IRCCS, Milan, Italy. MN-MCA1 cells were cultured in RPMI 1640 (Gibco) supplemented with 10% FCS. HEK293T Human embryonic kidney cells were kindly gifted by Dr Anna Schurich, King’s College London, London, UK. HEK293T cells were cultured in IMDM (Gibco) supplemented with 10% FCS. 3B-11 murine endothelial cells were obtained from ATCC. Cells were confirmed to be mycoplasma free using the MycoAlert Mycoplasma Detection Kit (Lonza).

### Formulation of KCL-HO-1i and off-target activity

KCL-HO-1i was generated by reacting 200 mg of mesoporphyrin IX (0.35 mmol, 1 eq., Merck, UK) with 576 mg of tin (II) pyrophosphate (1.4 mmol, 4 eq., Merck, UK) in a 50 mL round-bottom flask covered with tin foil. To this, 5 mL of glacial acetic acid was added, and the flask was flushed with nitrogen gas twice. The mixture was then stirred under reflux at 115°C. Once the temperature was stabilized, the reaction was exposed to air to introduce oxygen to the system, and then the reaction was left for 24 h. The mixture then cooled down to RT, quenched with 4 mL of HPLC grade water, filtered, and left to air dry for 10 min. The filtered solid was placed into another 50 mL flask with 3 mL of HPLC grade water and 0.48 mL of concentrated HCl and then left to stir for 30 min at 90 °C. The mixture was cooled down to RT and then was filtered and washed with cold water. After air-drying for 30 min, 125 mg of the product was obtained as a red powder (40% isolated yield) (Figure S2). The compound was fully characterized using a combination of liquid chromatography mass cytometry, high resolution mass spectrometry, and nuclear magnetic resonance techniques. Elemental analysis was performed by the London Metallomics Facility at King’s College London using a Perkin Elmer NexION 5000 inductively coupled plasma mass spectrometer (ICP-MS). Off-target activity of KCL-HO-1i was assessed at 10μM using the SafetyScreen44-panlabs panel (44 different enzymes and receptors) by Eurofins.

### *In vivo* PK studies

Pharmacokinetics studies were performed by Pharmacology Discovery Services (PDS) Taiwan, Ltd. as a contract research organization. A single dose of 25 µMol/Kg was administered to male ICR mice via the indicated route and blood aliquots (∼300 μL) were obtained via cardiac puncture from euthanised mice using tubes coated with lithium heparin at the indicated times. Blood samples were then gently mixed and centrifuged at 2,500 x *g* for 15 min at 4 °C within an hour of collection. Subsequently, the plasma samples were harvested and stored frozen at -70°C until further processing. The plasma samples underwent methanol (MeOH) precipitation and were analysed using LC-MS/MS. An Agilent Poroshell 120 EC-C18 column with dimensions of 2.7 μm (3.0 x 50 mm) was utilized for chromatographic separation. The mobile phase consisted of 0.2 % formic acid (FA) in water (solvent A) and 0.2 % FA in acetonitrile (ACN; solvent B), with a gradient from 10 % to 80 % (solvent B) over a 2.5-min period at a flow rate of 0.5 mL/min with a sample injection volume of 5 µL. The bioanalytical method involved utilizing mouse plasma as the sample matrix and 20 μL of sample into a well of a 96-well plate. Subsequently, 300 μL of 0.01 ng/μL oxybutynin internal standard (IS) in MeOH was added to each well, excluding double blank (00) and carryover. For the double blank well and carryover, 300 μL of MeOH was added. After vortexing for 1 min and centrifuging at 1000 x *g* for 5 min, 200 μL of supernatant was taken and mixed with 300 μL of MeOH/water (50/50) for LC-MS/MS analysis. Finally, the exposure levels (ng/mL) of KCL-HO1i in plasma samples were determined using LC-MS/MS.

### Tumor studies

MN-MCA1 sarcoma cells were orthotopically implanted into syngeneic C57Bl/6 mice for tumors. A total of 2.5 x 10^5^ cells in 100 μL RPMI were injected subcutaneously into female mice. In *MMTV-PyMT* mice, tumors arose spontaneously. When tumors became palpable, volumes were measured every 2-4 days using digital caliper measurements of the long (L) and short (S) dimensions of the tumor. Tumor volume was established using the equation Volume= (S^2^xL)/2. Tumor tissue for flow cytometry analyses was enzyme-digested to release single cells as previously described (31, 73). In brief, tissues were minced using scalpels, and then single cells were liberated by incubation for 60 min at 37 °C with 1 mg/mL Collagenase I from *Clostridium Histolyticum* (Sigma-Aldrich) and 0.1 mg/mL Deoxyribonuclease I (AppliChem) in RPMI. Released cells were then passed through a 70 μm cell strainer prior to staining for flow cytometry analyses. Viable cells were numerated using a hemocytometer with trypan blue (Sigma-Aldrich) exclusion. For drug treatments, drugs were freshly prepared on the day of injection and administered by intraperitoneal (i.p.) injection using a 26 G needle or orally administrated using a flexible, single use feeding needles (Cadence Science). Sn (IV) mesoporphyrin IX dichloride (SnMP; Frontier Scientific) was dissolved and administered as previously described (32). KCL-HO-1i was dissolved as per SnMP and administrated at 25µMol/kg/daily. 5-FU (Sigma-Aldrich) and gemcitabine (Sigma-Aldrich) were prepared fresh, dissolved in saline, and injected into mice i.p. using dosing regimens of 40 mg/kg/4 days and 6.6 mg/kg/7 days, respectively. Anti-mouse PD-1 (RMP1-14; Biolegend^®^) was administered at 12 mg/kg every 3 days. Immune-depleted mice were injected i.p. every 4 days, starting 48 h prior to the commencement of treatment, with 400 µg of anti-CD8α (53-6.7) (Biolegend^®^).

### *In vitro* derived macrophage polarization

Murine bone marrow (BM) was flushed from the femur and tibia of WT or *MMTV-PyMT* x *Hmox1*^fl/fl^ mice with (*Lyve1*^*Cre*^) or without (*Lyve1*^*WT*^) Cre using a syringe and needle. RBC were lysed using RBC lysis buffer (Roche). BM cells were plated in RPMI, 10% FCS, 1 x penicillin/streptomycin (Sigma-Aldrich), 10 ng/mL recombinant murine CSF-1 (Bio-Techne) at 1 x 10^6^ cells/well on 6 well plates for 72 h prior to subsequent polarization and downstream protein analyses. Murine IL-6 (R&D Systems) at 50 ng/mL was added where indicated in the figure legend at 50 ng/mL unless stated otherwise to generate LYVE-1^+^ HO-1^+^ macrophages (M_IL6_) (9).

### *In vitro* HO-1 activity assay

Rat and human HO-1 activity assays were developed by modifying previous assays described by Ryter et al (74), Braggins et al (75) and Yu et al (76). Microsomal fractions were utilized as a source of rat HO-1. Rat spleens were homogenized in (15 % w/v) ice-cold homogenizing buffer (50 mM Tris-HCl, pH 7.4, 0.25 M sucrose) using a motorized homogenizer (Potter-Elvehjem). An initial centrifugation of the homogenate took place at 10,000 x *g* for 20 min at 4 °C, and the supernatant was recovered and underwent further ultracentrifugation at 100,000 x *g* for 60 min at 4 °C. The 100,000 x *g* microsomal pellet was dissolved in an appropriate volume of homogenization buffer (100 mM KH_2_PO_4_, pH 7.8, 2 mM MgCl_2_) using a Teflon pestle to homogenize. For the human HO-1 activity assay, HEK293T cells were treated with 50 µM of hemin (Sigma-Aldrich) for 24 h to upregulate HO-1 expression. The cells were then harvested using enzyme-free cell dissociation buffer (Thermo Fisher) and washed three times at 1,000 x *g* in PBS to remove any residual hemin. On the last wash, the cell pellet was resuspended in homogenization buffer and sonicated using ultrasonic homogenizer (Sonics Vibra-cell). Protein concentration of the preparations was determined using the Pierce™ BCA Protein Assay Kit (Thermo Fisher Scientific) using the manufacturers’ protocol. Samples were stored at -80° C until use and stored no longer than 2 months. Biliverdin reductase for the assay was partially purified from liver cytosol. In brief, rat livers were perfused with ice-cold 0.9 % NaCl solution via the hepatic portal vein, tissue was then minced and washed with PBS to remove residual blood before homogenizing on ice with a Potter-Elvehjem homogeniser in 3 volumes of buffer containing 1.15 % KCl (w/v), 20 mM Tris-HCl pH 7.8. The homogenates were centrifuged first at 10,000 x *g* for 20 min at 4 °C and the supernatant taken and centrifuged at 100,000 x *g* for 1 h at 4 °C. Protein concentration of the final supernatant was determined by BCA assay, and the extract aliquoted and stored at -80 °C for no longer than 2 months. HO-1 activity was determined by spectrophotometrically quantifying bilirubin from microsomes at λ 464-600 nm. Activity assays contained 20 mM Tris-HCl (pH 7.4), homogenization lysate or purified microsomes (0.75 mg), partially purified biliverdin reductase (1.5 mg), 2 mM glucose-6-phosphate (Sigma-Aldrich) in stabilising buffer (20 mM Tris-HCl, 2 mM MgCl_2_), 1 Unit of glucose-6-phosphate dehydrogenase in stabilizing buffer, 25 µM hemin (Sigma-Aldrich) in a volume of 750 µL. Vehicle and KCL-HO-1i was then spiked into the assay and samples were briefly vortexed and transferred to a shaking incubator for 15 min, 600 rpm at 37 °C. Reactions were initiated by the addition of 1 mM NADPH in stabilizing buffer and transferred to the shaking incubator for 60 min in the dark. Bilirubin was extracted by adding one volume of chloroform and vortexing for 30 s, followed by centrifuging at 15,000 x *g* for 10 min. The organic layer was recovered and measured using a Chirascan Plus split-beam spectrophotometer (Applied Photophysics) in a temperature-controlled chamber held at 23 °C. Readings were obtained with a bandwidth of 2 nm and time-per-point of 0.5 s. Bilirubin was calculated assuming the reported molar extinction coefficient in chloroform (40 mM /cm^-1^). One enzyme unit was defined as the amount of enzyme that oxidizes 1 µmol of D-glucose 6-phosphate in the presence of NADP at pH 7.4 at 25 °C.

### Western blot

Cells were lyzed and sodium dodecyl sulphate polyacrylamide gel electrophoresis (SDS-PAGE)/western blots were conducted as previously described (3). In brief, cells were lyzed in the well using Western blot lysis buffer 0.1M Tris-hydrochloride pH 6.8, with 20 % glycerol and 4% SDS containing 1X protease and phosphatase inhibitor cocktail (Thermo Fisher Scientific). All tubes were heated at 95 °C for 15 min to break down DNA. Protein concentration was then determined using the Pierce™ BCA Protein Assay Kit (Thermo Fisher Scientific) using the manufacturers’ protocol. Samples were then run under reducing conditions using β-mercaptoethanol on 12% bis-tris SDS-PAGE gels alongside a SeeBlue™ Plus2 pre-stained maker (Thermo Fisher Scientific). SDS-PAGE gels were then transferred onto polyvinyl-difluoride (PVDF) membranes which were subsequently blocked in 100 mM Tris pH7.4, 140 mM NaCl, 0.1% Tween 20 (Sigma-Aldrich) (TBS-T) containing 5% skimmed milk at RT for 1 h. Primary antibodies were incubated O.N. at 4 °C and secondary antibodies for 1 h at RT. Wash steps to remove unbound antibodies were 3 x 20 min in TBS-T. The following primary antibodies were used: rabbit anti-β-actin 1:5,000 (ab8227, Abcam) and rabbit anti-HO-1 1:1,000 (10701-1-AP, Proteintech). These antibodies were detected using goat anti-rabbit immunoglobulins-horse radish peroxidase (HRP) secondary antibody 1:2,000 (Agilent Dako). Protein bands were detected using Luminata™ Crescendo Western HRP substrate (Millipore) and chemiluminescence was captured by Fusion Solo system (Vilber Lourmat, Eberhardzell, Germany) and were analyzed with ImageJ software.

### Immunofluorescence

Mouse tumor tissue was fixed for 18 h RT in 4% paraformaldehyde, followed by 12 h dehydration in 30% sucrose (Sigma-Aldrich) prior to embedding in OCT (Optimal Cutting Temperature, VWR chemicals) and snap freezing absolute ethanol and dry ice. Sections (10 µm) from the embedded tumors were placed onto microscope slides (VWR International) and were incubated further in 4% paraformaldehyde in DPBS for 10 min at RT prior to washing in TBS-T. Sections from formalin-fixed paraffin-embedded (FFPE) human in invasive ductal carcinoma tissue tumor tissue was acquired from the King’s Health Partners Biobank. FFPE Sections were deparaffinized in xylenes (3 washes x 5 min) and rehydrated in an ethanol/water gradient series: 100% EtOH (2 washes x 5 min), 95% EtOH (2 washes x 5 min), and finally water (2 washes x 5 min). Epitopes were unmasked using 20 mg/mL of Proteinase K (25530049; Thermo Fisher Scientific) and incubated for 3 min at RT. The slides were then washed with TBS-T (3 washes x 3 min). Mouse and human slides were blocked using TBS-T, 10% donkey serum (Sigma-Aldrich), 0.2% Triton X-100 (Sigma-Aldrich) for 1 hour at RT. Immunofluorescence staining was performed as previously described (3). For mouse tissues, antibodies against the following targets and their dilutions were used as follows: CD31 1:100 (AF3628 R&D Systems), F4/80 1:100 (C1:A3-1, Bio-Rad), HO-1 1:100 (10701-1-AP, Proteintech), CD3 1:100 (SP7, Abcam), CD8 (EPR21769, Abcam). For human tissues, antibodies against the following targets and their dilutions were used as follows: CD31-AlexaFluor^®^ 647 10 µg/mL (clone: JCC/70A, ab215912, Abcam), CD68-AlexaFluor^®^ 594 0.5 µg/mL (clone: KPI, SC-20060, Santa Cruz) and HO-1 1:100 (10701-1-AP, Proteintech). Unconjugated primary antibodies were detected using antigen specific donkey IgG, used at 1:200 AlexaFluor^®^ 488 anti-rabbit IgG, AlexaFluor^®^ 488 anti-goat IgG, AlexaFluor^®^ 568 anti-rabbit IgG, AlexaFluor^®^ 568 anti-goat IgG, AlexaFluor^®^ 647 anti-rabbit IgG, AlexaFluor^®^ 647 anti-rat IgG, AlexaFluor^®^ 532 anti-rabbit IgG (Thermo Fisher Scientific). Nuclei were either stained using 1.25 μg/mL 4’,6-diamidino-2-phenylindole,dihydrochloride (DAPI) (Thermo Fisher Scientific) or 500 nM SYTO^®^ Green Fluorescence Nucleic Acid stain (S7575, Thermo Fisher Scientific). Images were acquired using a Nikon Eclipse Ti-E Inverted spinning disk confocal and the Nikon SoRa. Images were analyzed using the NIS-Elements software.

### H&E staining and histopathology scoring

Tissue sections (3µm) were cut from FFPE tissues from the brain, lung, heart, kidney, and liver of treated animals and mounted onto Plus^+^ Frost slides (Solmedia). Sections were incubated at 60 °C for 1h before dewaxing and H&E stained using the Tissue-Tek DRS 2000 automated slide stainer (Leica Biosystems). Once stained, a cover slip was applied using the ClearVue™ Coverslipper (Thermo Fisher Scientific). H&E-stained tissue sections were examined by a Royal College of Veterinary Surgeons recognized specialist in veterinary pathology. Tissue sections were initially examined unblinded and then re-evaluated blinded to confirm the significant findings (77). Findings in the tissue were scored using a non-linear semiquantitative grading system from 0 to 5 where 0 = no significant change and 5 = whole organ or tissue affected (78). Where grading was not appropriate, findings were scored ‘present’ (P) if seen. In addition, an approximate number of lung metastases was recorded in parentheses after P if present (78).

### Bioluminescence imaging

For assessing luciferase expression in HO-1^Luc/eGFP^ mice *in vivo*, mice were injected i.p. with 3 mg XenoLight D-luciferin (PerkinElmer) diluted in sterile DPBS 10 min prior to imaging. For whole-body imaging, animals were anesthetized and placed in the *in vivo* Imaging System (IVIS^®^) Lumina Series III (PerkinElmer). For imaging luciferase expression in different tissues, mice were sacrificed, and the dissected tissues were then collected and imaged. To quantify luminescence, a region of interest (ROI) was drawn around a specific area and total photon flux (PF) (photon/second; p/s) was measured. All data was analyzed using the Living Image Software (PerkinElmer).

### T-cell isolation

For isolating murine T-cells, spleens were excised from WT C57Bl/6 mice and placed in RPMI 1640, 10% FCS, 20 µM 2-mercaptoethanol, 1X penicillin/streptomycin (Sigma-Aldrich). Spleens were crushed through a 70 µm pore strainer and washed through using RPMI 1640. Liberated splenocytes were centrifuged at 500 x *g* for 3 mins and the cell pellet was re-suspended in 1 mL of red blood cell lysis buffer (Roche) for 2 mins at RT. Cells were then centrifuged at 500 x *g* for 8 mins and the pellet was resuspended in RPMI 1640. Live cells were numerated using Trypan blue (Sigma-Aldrich) exclusion on a hemacytometer. CD3^+^ T-cells were purified using the CD8a^+^ T-cell isolation Kit, mouse (Miltenyi Biotec) and isolated using a QuadroMacs separator and LS columns (Miltenyi Biotec) according to the manufacturers’ instruction. T-cells were resuspended in T-cell culture media that was further supplemented with 2 ng/mL recombinant murine IL-2 (Bio-Techne) and purified CD8^+^ T-cells were plated at a density of 0.1x10^6^ cells/well in 200 µL onto a high binding 96-well plate (Corning) that had been pre-coated O.N. with a mix of anti-mouse CD3ε (145-2C11, 5 µg/mL) and anti-mouse CD28 (37.51, 3 µg/mL) antibodies in sterile PBS (100 µL/well) at 4°C. After 48 h CD8^+^ T-cells were transferred to a fresh uncoated plate and rested for at least 48 h before being numerated and used for down-stream *in vitro* assays. Murine CD8^+^ T-cells were seeded were seeded at a density of 0.3x10^6^ cells/well in 200 µL onto a 96-well plate (Sigma-Aldrich). Then CD8^+^ T-cells were exposed to 25 µM KCL-HO-1i, 5 μM hemin (Sigma-Aldrich), 20 μM iron (II) chloride (Sigma-Aldrich), 5 μM biliverdin hydrochloride (Sigma-Aldrich), or a gas mix of 250 ppm CO, 5% CO_2_, N_2_ balance (BOC) in a hypoxia incubator chamber (StemCell Technologies) for 16 hours. The cells were collected and stained for flow cytometry analysis.

### *In vitro* Pv nest transwell assay

This assay was adapted from (9). Transwell inserts (8 µm pores; Corning) were coated with Basement Membrane Extract (Cultrex) diluted 1:100 in RPMI for 1 h at RT. Excess Basement Membrane Extract was aspirated and 2 x 10^4^ 3B-11 endothelial cells were seeded onto the apical side of the transwell insert in RPMI supplemented with 10% FCS and left to attach for 24 h. Media was removed, the whole plate inverted and 10^5^ M_(0)_ or M_(IL-6)_ BMDMs were seeded onto the basolateral side of the transwell membrane in RPMI supplemented with 10% FCS and left to attach for 2 h at 37ºC. Subsequently, the plate was reinverted to its original position and RPMI supplemented with 10% FCS, 10 ng/mL M-CSF with or without 10 ng/mL IL-6 added to the apical and basolateral space. After cells were left to interact for 24 h at 37ºC, 4 x 10^5^ CD8^+^ T cells (which had been prior incubated on anti-CD3 and -CD28 coated plated to develop effector function) were added to the inserts in RPMI, 10% FCS in the presence of absence of 25 µM KCL-HO-1i. After 16 h, migrated cells were collected from the well and stained for flow cytometry analysis and quantification with AccuCheck counting beads (Thermo Fisher Scientific).

### Flow cytometry and cell sorting

Flow cytometry was performed as previously described (32). Fc receptors were blocked using 1 µg/mL CD16/32 (2.4G2; Tonbo Biosciences) incubated for 30 min on ice. The following antibodies against the indicated antigen were purchased from Thermo Fisher Scientific and were used at 1 µg/mL unless stated otherwise: CD3ε Alexa Fluor^®^ 488, APC, PE, PE-Cy7 (145-2C11) and Brilliant Violet (BV)421 (17A2; Biolegend^®^), CD4 APC, FITC and PE (RM4-5), CD8α BV421, BV711 and FITC (53-6.7; Biolegend^®^), CD8β FITC and eFluor^®^450 (H35-17.2), CD11b BV510 (M1/70; Biolegend^®^), CD11c APC (N418) and FITC (N418; Biolegend^®^), CD19 BV421, APC (6D5; Biolegend^®^) and FITC (1D3/CD19; Biolegend^®^), CD31 FITC, PE, PE-Cy7 (390) and BV510 (MEC 13.3; BD biosciences), CD44 BV510 (IM7; Biolegend^®^), CD45 BV605 (30-F11; Biolegend^®^), APC, APC-eFluor^®^ 780 (30-F11), BV510 and BV785 (30-F11; Biolegend^®^), CD62L APC (MEL-14; Biolegend^®^), CD90.1 eFluor^®^ 450 (HIS51) and BV510 (OX-7; Biolegend^®^), CD69 PE (H1.2F3: Biolegend®), CD90.2 eFluor^®^ 450 (53-2.1) and BV510 (53-2.1; Biolegend^®^), CD103 BV421 (2E7: Biolegend®), CD206 APC (FAB2535A; Bio-Techne), FITC and BV785 (C068C2; Biolegend^®^), F4/80 APC, APC-eFluor^®^ 780, PE and BV605 (BM8), BV421 and FITC (BM8; Biolegend^®^), Gr-1 FITC (RB6-8C5; Biolegend^®^), IFN-γ PE (XMG1.2), LAG3 PerCP-Cy5.5 (C9B7W: Biolegend®), Ly6C APC-eFluor^®^ 780, BV421, PE and FITC (HK1.4; Biolegend^®^), Ki69 APC-eFluor®780 (SolA15), Ly6G FITC (1A8; Biolegend^®^), LYVE-1 Alexa Fluor^®^ 488, PE, PE-Cy7 (ALY7) and APC (FAB2125A; Bio-Techne), MHCII PE (M5/114.15.2), BV421, BV510 and FITC (M5/114.15.2; Biolegend^®^), NK1.1 APC (PK136), PD1 BV510 (29F.1A12: Biolegend®) and TIM3 BV421 (RMT1-4: Biolegend®). Positive stains were compared to fluorescence minus one (FMO) control. Intracellular stains were performed as previously described (32) using the Foxp3 Transcription factor staining buffer set (Thermo Fisher Scientific). Dead cells and red blood cells were excluded using 1 µg/mL 7-amino actinomycin D (7AAD; Sigma-Aldrich) or Fixable Viability Dye eFluor^®^ 780 or Near-IR Dead cell staining kit (Thermo Fisher Scientific) alongside anti-Ter-119 PerCP-Cy5.5 or APC-eFluor^®^ 780 (Ter-119, Thermo Fisher Scientific). Data were collected on a BD FACS Canto II (BD Biosciences) or CytoFLEX LX Flow (Beckman Coulter). Data was analyzed using FlowJo software (BD biosciences). Immune cells (CD45^+^) were separated based upon the following surface characteristics: CD11c^+^F4/80^-^ (dendritic cells), CD11b^+^F4/80^hi^ (macrophages), F4/80^-/lo^Ly6G^-^Ly6C^+^ (monocytes), CD11b^+^Ly6G^+^ (neutrophils), NK1.1^+^ (NK/NKT-cells), CD3ε^+^ (T-cells), CD3ε^+^CD4^+^ (CD4^+^ T-cells), CD3ε^+^CD8α/β^+^ (CD8^+^ T-cells), CD3ε^-^CD19^+^ (B cells). Cancer associated fibroblasts (CAFs) were identified as CD45^-^ Thy1^+^ cells and tumor cells were identified as CD45^-^Thy1^-^CD31^-^. Cells sorting was performed using the BD FACSAria Fusion cell sorter (BD Biosciences).

### Assessment of liver function

Mice were humanely sacrificed by overdose of pentobarbital. Blood was collected from mice by cardiac puncture in Microvette® CB300 blood collection tubes (KMIC-SER, Microvette®). Plasma was generated by centrifugation of the collection tubes were at 1,000 x *g* for 10 min at 4°C, and plasma was collected from the sample and stored at -80°C until use. Liver function was assessed in the plasma by measuring Aspartate aminotransferase (AST) and Alanine transferase (ALT) liver enzymes through ELISA using Mouse AST ELISA Kit (ab263882, Abcam) and Mouse ALT ELISA Kit (ab282882, Abcam) according to the manufacturer’s instructions.

### Transcriptomic data acquisition

Tumor tissues were preserved in RNAlater (Thermo Fisher Scientific) and stored -80 °C until use. mRNA was purified using the RNeasy Mini kit (74104, Qiagen^®^) according to the manufacturer’s instructions. The purity of the isolated mRNA was assessed using a NanoDrop™ 2000 spectrophotometer (Thermo Fisher Scientific) and the quality and integrity using an Agilent 2100 Bioanalyzer (Agilent Technologies). OD280/OD260 absorbance ratio >1.9 and RNA Integrity Number >9 was obtained. Next generation sequencing (NGS) was performed by GENEWIZ from Azenta Life Sciences.

### Transcriptomic data analysis

The read quality of all the samples was checked using *FastQC v0.11.9*. The alignment was conducted using the *STAR aligner v2.7.9a*. The count table was generated as TPM counts using the *quant* function from *Salmon v1.9.0*. The transcript level counts were summarized to Gene level counts using the *tximport* package in R. Differential Analysis was done using the *DeSeq2* package in R.

The differentially upregulated gene list in all the five treatments (5-FU, gemcitabine, KCL-HO-1i, 5-FU/KCL-HO-1i and gemcitabine/KCL-HO-1i) was generated by comparing to the vehicle treated samples using a logFC cutoff of 0.6 and p-value of 0.05. To identify the commonly upregulated genes among KCL-HO-1i treated cohorts, we took the intersection of upregulated genes in KCL-HO-1i, 5-FU/KCL-HO-1i and gemcitabine/KCL-HO-1i treatments using the *venn* package in R. Similarly, the commonly upregulated genes among chemotherapy-based therapies were identified from the intersection of upregulated genes in 5-FU, gemcitabine, 5-FU/KCL-HO-1i and gemcitabine /KCL-HO-1i treatments. The upregulated genes were also analyzed using the *groupGO* function from the *clusterProfiler* package in R. For cellular compartmentalization of the commonly upregulated genes in KCL-HO-1i and/or chemotherapy treatment groups, we used the GO:CC ontology and identified the genes enriched in the extracellular space (secreted proteins). Next to understand the biomolecular functions of the common extracellularly enriched genes in KCL-HO-1i treated cohorts, we used the GO:MF and GO: BP ontology to identify the top molecular function and biological process pathways.

The differentially upregulated gene list for sorted CD206^+^LYVE1^+^ TAMs from KCL-HO-1i treated tumors of PyMT mice was generated by comparing to the vehicle treated tumors using a logFC cutoff of 0.6 and adjusted p-value of 0.05. To compare if the differentially expressed gene profile was similar to any of the TAM subsets, we generated upset plots using the differentially upregulated genes for all TAM subsets from our previously published work using the *UpSetR* package in R (18). To understand the biomolecular functions of the enriched genes in the KCL-HO-1i treated cohort, we used the GO:BP ontology to identify the top biological process pathways.

### Analysis of scRNAseq transcriptomic datasets

Publicly available scRNAseq datasets were re-analyzed to investigate the quantitative association between LYVE-1^+^ PvTAMs and infiltrating CD8^+^ T-cells. For this analysis, scRNAseq data from TAMs in a *MMTV-PyMT* tumor (GSE160641) and human breast adenocarcinoma (GSE161529 and GSE176078) were used. All the datasets were processed using the 10X Genomics platform. Murine TAM scRNAseq data was clustered and annotated to identify the LYVE-1^+^ TAMs using the standard integration pipeline (https://satijalab.org/seurat/articles/integration_introduction) in *Seurat v5* package. We integrated the two human datasets using the *Scalex* package in python (https://scalex.readthedocs.io/en/latest/index.html). Altogether, this dataset has 153,087 cells with 15,326 myeloid cells, 1,996 endothelial cells and 25,899 T-cells, identified by the lineage genes *CD68, PECAM1* and *CD3D* respectively. We next did a label transfer from the murine myeloid cluster to identify the LYVE-1^+^ TAM cluster using the *Seurat v5* package (https://satijalab.org/seurat/articles/integration_mapping). The gene symbols in the murine dataset were converted to human symbols before building the integrated reference. Therefore, we could quantify the total number of LYVE-1^+^ TAMs in each of the human sample (30 in total across the integrated dataset) alongside endothelial cells (*PECAM*^+^), T-cells (CD3D^+^) and CD8^+^ T-cells (CD8A^+^) for correlative analyses. We filtered out all the samples which didn’t contain either LYVE-1^+^ TAMs or CD8^+^ T-cells and excluded an outlier with an apparent exceedingly high LYVE-1^+^ TAM abundance.

### Statistics

Normality and homogeneity of variance were determined using a Shapiro-Wilk normality test and an F-test respectively. Statistical significance was then determined using a two-sided unpaired Students *t* test for parametric data, Mann-Whitney or one-way ANOVA tests followed by Tukey’s multiple comparison tests were performed. A Welch’s correction was applied when comparing groups with unequal variances. Significance of correlations were assessed using Pearson’s correlation test. Statistics was performed using GraphPad Prism 9 software Statistical analysis of tumor growth curves was performed using the “CompareGrowthCurves” function of the statmod software package (79).

### Study approval

All experiments involving animals were approved by the Animal and Welfare and Ethical Review Board of King’s College London and Home Office UK. *In vivo* experiments were performed under Project Licence PP3601022. For the PK studies, animal housing, experimentation, and disposal was conducted in strict adherence to the Guide for the Care and Use of Laboratory Animals: Eighth Edition (National Academy Press, Washington, D. C., 2011) within an AAALAC-accredited laboratory animal facility. The IACUC at Pharmacology Discovery Services Taiwan, Ltd., reviewed and approved the animal care and use protocol. Human breast adenocarcinoma tissue was obtained with informed consent under ethical approval from the King’s Health Partners Cancer Biobank (REC reference 12/EE/0493).

## Supplementary Material

Supplementary Material

## Data Availability

Bulk RNAseq data generated in this study is available GSE248272 (bulk tumor) and GSE287874 (isolated LYVE-1^+^ TAMs). All other data can be made available on request to the corresponding authors.

## One sentence summary

This study describes the development of an orally bioavailable small molecule heme oxygenase inhibitor that targets immunosuppressive perivascular macrophages in the tumor microenvironment, offering an immunotherapeutic strategy to disrupt macrophage-mediated suppression of T-cell responses in cancer and synergizing with chemotherapy drugs to inhibit tumor growth.
