# Supplementary material for: An oral heme oxygenase inhibitor targets immunosuppressive perivascular macrophages in preclinical models of cancer
Source: Sci Transl Med. Author manuscript; Available in PMC 2025 Aug 27. (PMC7618036; doi:10.1126/scitranslmed.ads3085)
Supplement: Supplementary Material [file EMS207789-supplement-Supplementary_Material.pdf]

Supplementary Materials

An oral heme oxygenase inhibitor targets immunosuppressive perivascular macrophages in preclinical models of cancer

Meriem Bahri, Taha Al-Adhami, Emre Demirel, Jit Sarkar, Karen T. Feehan, Joanne E. Anstee, Tik Shing Cheung, Dominika Sosnowska, Chloé A. Woodman, William Macmorland, Dorothy D. Yang, James Rosekilly, Renee Gitsaki-Taylor, Cheryl E. Gillett, Cheryl L. Scudamore, James Spicer, Khondaker Miraz Rahman and James N. Arnold

Receptor/enzyme	Species	Inhibition (%)
Cholinesterase	Human	9
Cyclooxygenase-1	Human	-2
Cyclooxygenase-2	Human	10
Monoamine oxidase	Human	1
Phosphodiesterase 3A	Human	61
Phosphodiesterase 4D2	Human	28
Protein tyrosine kinase LCK	Human	-18
Adenosine A_{2A}	Human	-3
Adrenergic α_{1A}	Human	0
Adrenergic α_{2A}	Human	9
Adrenergic β_1	Human	-9
Adrenergic β_2	Human	1
Androgen (testosterone)	Human	5
Calcium channel L-type	Rat	3
Cannabinoid 1	Human	2
Cannabinoid 2	Human	-2
Cholecystokinin 1	Human	19
Dopamine D_1	Human	8
Dopamine D_{2s}	Human	15
Endothelin A	Human	-5
GABAA	Rat	13
Glucocorticoid	Human	27
Glutamate	Rat	8
Histamine H_1	Human	10
Histamine H_2	Human	-16
Muscarinic M_1	Human	-9
Muscarinic M_2	Human	-4
Muscarinic M_3	Human	8
Nicotine Acetylcholine α4β2	Human	0
Opiate δ_1	Human	10
Opiate κ	Human	3
Opiate μ	Human	-1
Potassium Channel K_A	Rat	-11
Potassium Channel hERG	Human	-3
Serotonin 1A	Human	16
Serotonin 1B	Human	-10
Serotonin 2A	Human	-2
Serotonin 2B	Human	18
Serotonin 3	Human	14
Sodium Channel Nav1.5	Human	5
Transporter, Dopamine	Human	0
Transporter, Norepinephrine	Human	20
Transporter, Serotonin	Human	5
Vasopressin 1A	Human	1

Table S1. Analysis of off-target specificity of KCL-HO-1i
Assessment of the off-target activity of KCL-HO-1i (10μM) against 44 different receptors/enzymes. The table presents the % inhibition of enzyme activity or radioligand binding (to receptors) for each target. A significant response is considered >50% inhibition in the above assays (highlighted in orange).

Tissue	Pathology noted		1	2	3	4	5	6	7	8	9	10	11	12	13	14	15	16	17	18	19	20	Mouse
			Vehicle	Vehicle	Vehicle	Vehicle	Vehicle	KCL-HO-1i	KCL-HO-1i	KCL-HO-1i	KCL-HO-1i	KCL-HO-1i	Gemc	Gemc	Gemc	Gemc	Gemc	KCL-HO-1i Gemc	KCL-HO-1i Gemc	KCL-HO-1i Gemc	KCL-HO-1i Gemc	KCL-HO-1i Gemc	Treatment
Brain			0	0	0	0	0	0	0	0	0	0	0	0	0	0	0	0	0	0	0	0	
Heart	Mononuclear inflammatory cell infiltrate, focal	artia	0	0	0	0	0	0	0	0	0	0	0	0	0	0	0	1	0	0	0	0	
Lungs	Metastases		P(20+)	P(1)	0	0	P (10+)	P(30+)	P(10+)	P(30+)	0	P(1)	0	0	0	0	P(2)	0	0	0	0	0	
Lungs	Mononuclear inflammatory cell infiltrate, multifocal	perivascular	0	0	0	0	0	0	0	1	2	0	0	0	0	1	0	3	2	3	3	3	
Lungs	Mixed inflammatory cell infiltrate, focal	alveolar	0	0	0	0	0	0	0	0	0	0	0	0	0	0	0	2	1	1	2	2	
Lungs	Foamy macrophages	alveolar	0	0	0	0	0	0	0	0	0	0	0	0	0	0	0	1	0	0	0	0	
Kidney	Tubular basophilia		0	0	0	0	0	0	0	0	0	1	0	0	0	0	0	0	0	0	0	0	
Liver	Inflammatory cell foci		1	1	0	1	1	0	0	0	1	1	0	1	1	1	1	0	0	0	0	1	
Liver	Increased mitoses	Hepatocellular	1	0	2	1	1	3	3	0	0	1	0	2	0	0	1	0	0	0	0	0	
Liver	Pigmented macrophages, focal	Subcapsular	0	0	0	0	0	1	0	0	0	0	0	0	0	0	0	0	0	0	0	0	
Liver	Focal necrosis		0	0	0	0	0	0	0	1	0	0	0	0	0	1	0	0	0	0	0	0	

Table S2. Histopathological evaluation of organs from mice treated with KCL-HO-1i and/or gemcitabine. Mice were dosed p.o. with KCL-HO-1i (25 µMol/kg/day) and/or gemcitabine (6.64 mg/kg/7days) or vehicle in *MMTV-PyMT* mice bearing established tumors for 21 days. At the end of treatment, mice were sacrificed and the brain, lungs, liver, kidney, heart were harvested for histological examination. H&E-stained tissue sections from each mouse and tissue were scored by a pathologist. The table presents all identified histological observations for those tissues where features were noted. Scores are a non-linear, semi-quantitative grading system from 0 to 5, where 0 = no significant change and 5 = whole organ or tissue affected for each observation. P denotes ‘present’.

		End of treatment										30 days post end of treatment										
		1	2	3	4	5	6	7	8	9	10	1	2	3	4	5	6	7	8	9	10	Mouse
Pathology noted		Vehicle	Vehicle	Vehicle	Vehicle	Vehicle	KCL-HO-1i Gemc	KCL-HO-1i Gemc	KCL-HO-1i Gemc	KCL-HO-1i Gemc	KCL-HO-1i Gemc	Vehicle	Vehicle	Vehicle	Vehicle	Vehicle	KCL-HO-1i Gemc	KCL-HO-1i Gemc	KCL-HO-1i Gemc	KCL-HO-1i Gemc	KCL-HO-1i Gemc	Treatment
Mononuclear inflammatory cell infiltrate, multifocal	vascular/perivascular	0	0	0	0	0	3	3	1	1	2	0	0	0	1	0	0	2	0	1	1	
Mixed inflammatory cell infiltrate, focal	peribronchiolar	0	0	1	0	0	0	0	0	0	0	0	0	0	0	0	0	0	0	0	0	
Mononuclear inflammatory cell infiltrate, multifocal	alveolar	0	0	0	0	0	2	1	0	0	0	0	0	0	0	0	0	0	0	0	0	
Focal fibrosis	alveolar	0	0	0	0	0	2	0	0	0	0	0	0	0	0	0	0	0	0	0	0	
Macrophage accumlation	alveolar	0	0	0	0	0	0	0	0	0	0	0	0	0	0	0	0	0	0	1	0	

Table S3. Histopathological evaluation of lungs from mice treated with KCL-HO-1i and/or gemcitabine. Non-tumor bearing C57Bl/6 mice were dosed p.o. with KCL-HO-1i (25 µMol/kg/day) and gemcitabine (6.64 mg/kg/7days) or vehicle for 21 days. Lungs were then excised from 1 cohort of mice at treatment cessation and a second 30 days after treatment cessation. Lung sections were H&E stained and assessed by a pathologist. The table presents all identified histological observations. Scores are a non-linear, semi-quantitative grading system from 0 to 5, where 0 = no significant change and 5 = whole organ or tissue affected for each observation.

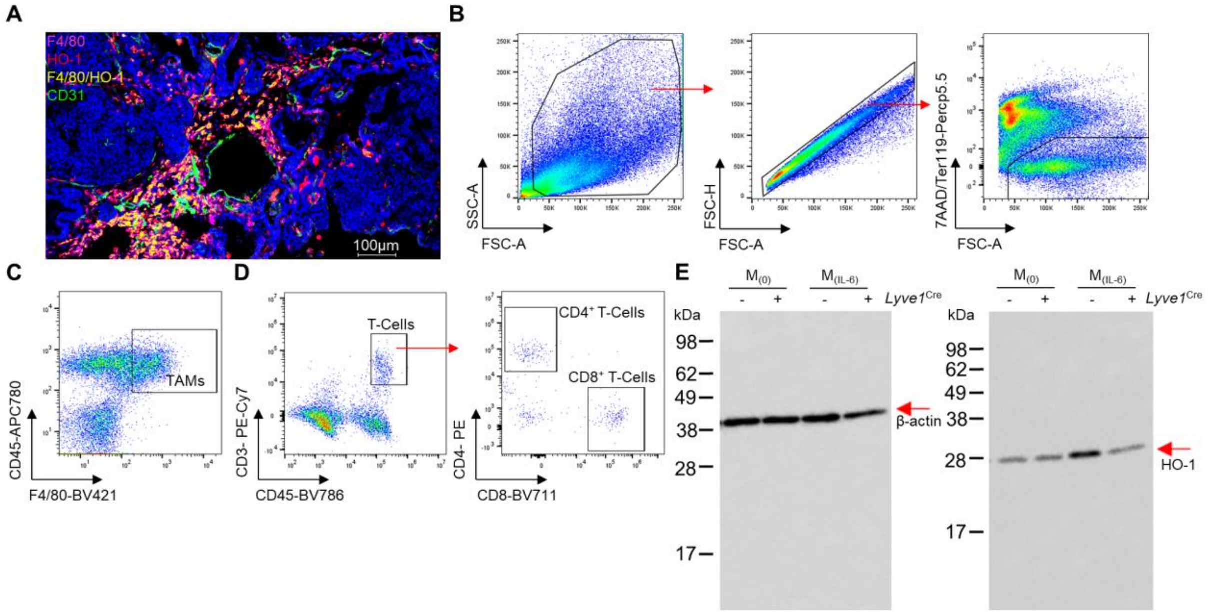

Figure S1. Analysis of the TME and PvTAM expression of HO-1
(A) A representative image of a frozen section of *MMTV-PyMT* tumor stained with DAPI (nuclei; blue) and antibodies against F4/80 (magenta), HO-1 (red), CD31 (green). Colocalizing pixels for F4/80 and HO-1 are shown in yellow. Scale bar is 100 µm.
(B-D) Representative gating strategy for identifying live (7AAD$^-$) tumoral single cells **(B)**, TAMs **(C)** and CD4$^+$ and CD8$^+$ T-cells **(D)** from an enzyme-digested *MMTV-PyMT* tumor. **(E)** Full western blot images from data presented in Figure 1 probing for HO-1 (right) and β-actin (left) expression in BMDMs under $M_{(0)}$ (CSF-1 alone) and IL-6 polarization ($M_{(IL\text{-}6)}$) conditions from mice carrying the *Hmox1*$^{fl/fl}$ allele with and without *Lyve1*^{Cre}.

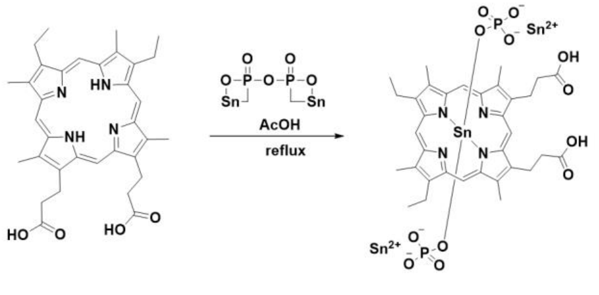

Mesoporphyrin IX **KCL-HO-1i**

Figure S2. Chemical synthesis of KCL-HO-1i. KCL-HO-1i was generated from mesoporphyrin IX by refluxing with tin (II) pyrophosphate in acetic acid. The final molecular structure was confirmed using a combination of liquid chromatography mass spectrometry, high resolution mass spectrometry, nuclear magnetic resonance and elemental analysis techniques as outlined in Materials and Methods.

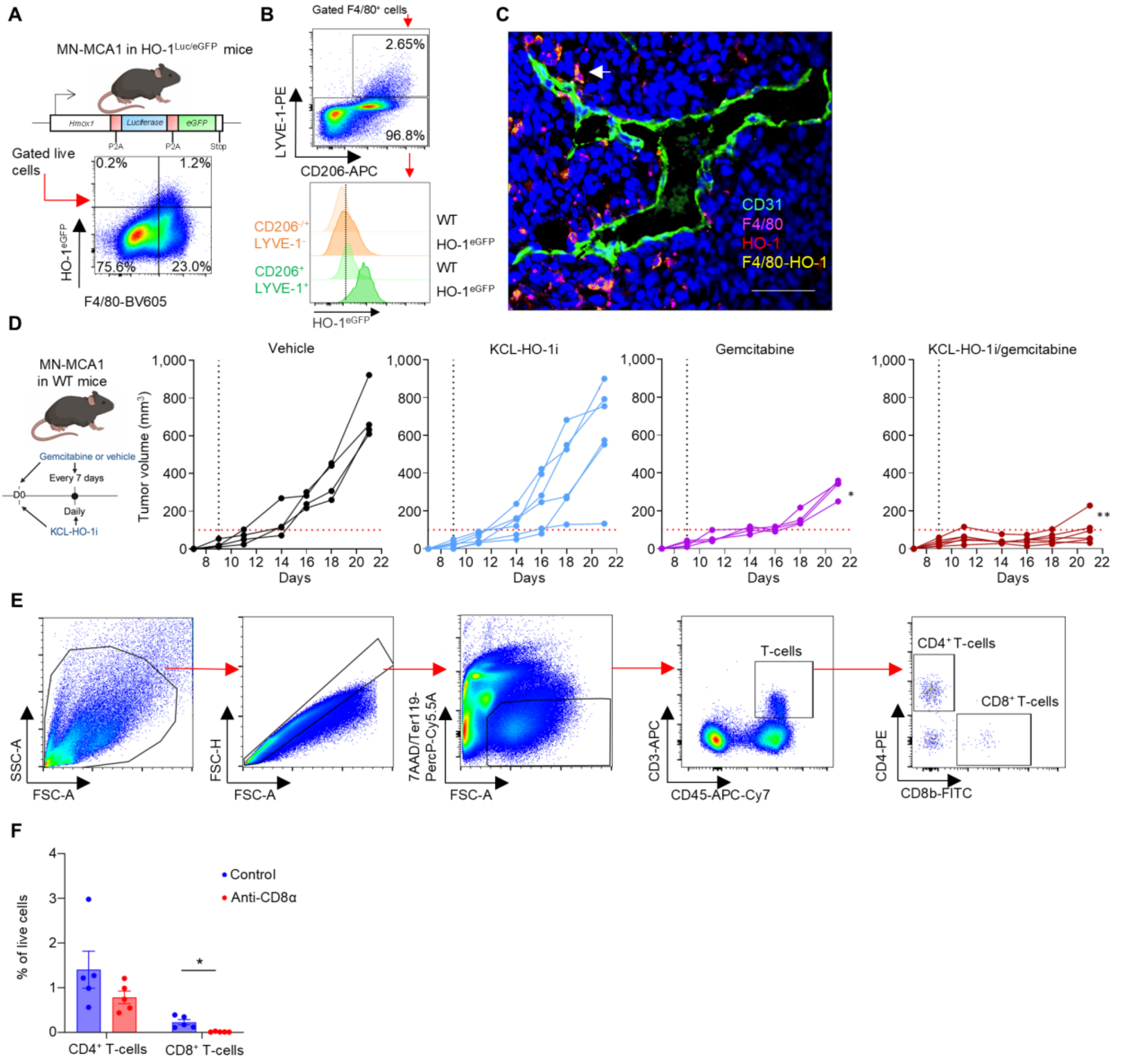

Figure S3. KCL-HO-1i synergizes with gemcitabine in MN-MCA-1 sarcoma tumors. (A-C) Schematic depicting the MN-MCA1 sarcoma cells subcutaneously injected in HO-1$^{Luc/eGFP}$ mice (top panel) and representative dot plots of FACs-gated live (7AAD^{-}) cells from enzyme-dispersed MN-MCA1 tumors separated based on their respective expression of HO-1 (eGFP) and F4/80 (bottom panel) **(A)**, and FACs-gated live (7AAD^{-}) TAM (F4/80^{+}) separated based on their respective expression of LYVE-1 and CD206 (top panel) and histograms for their expression of HO-1(eGFP) alongside histograms showing background fluorescence in tumors taken from WT mice using the same gating (bottom panel) **(B)**. **(C)** Representative image of a frozen section of MN-MCA1 sarcoma tumor stained with DAPI (nuclei; blue) and antibodies against F4/80 (magenta), HO-1 (red), CD31 (green). Colocalizing pixels for F4/80 and HO-1 are shown in yellow. White arrow highlights an example F4/80/HO-1 PvTAM. Scale bar denotes 50 µm. **(D)** Schematic representing the i.p. dosing strategy for KCL-HO-1i

(25 μMol/kg/day) or gemcitabine (6.6 mg/kg/7 days) and/or vehicle in C57BL/6 mice bearing established MN-MCA1 tumors (left) and the individual tumor growth curves for the respective treatment (right). Dashed black lines mark the start of treatment (day 0) and horizontal red line marks 100 mm^3 and solid lines represent individual tumors and mice. **(E-F)** Flow cytometry gating strategy for tumor infiltrating $CD8^+$ ($CD3^+$ $CD8\beta^+$) and $CD4^+$ ($CD3^+$ $CD4^+$) T-cells from an enzyme-dispersed *MMTV-PyMT* tumor (**E**) and quantification of tumor infiltrating $CD8^+$ and $CD4^+$ T-cells from *MMTV-PyMT* mice treated with or without anti-CD8α antibodies (n=5, each dot represents an individual tumor and mouse), growth curves shown in Figure 1H **(F)**. Images in panel **(A)** and **(D)** were created using *BioRender*. Bar charts represent the mean, error bars SD, and the dots show individual data points from individual tumors and mice. Line charts display the mean and SEM. * $P<0.05$, ** $P<0.01$.

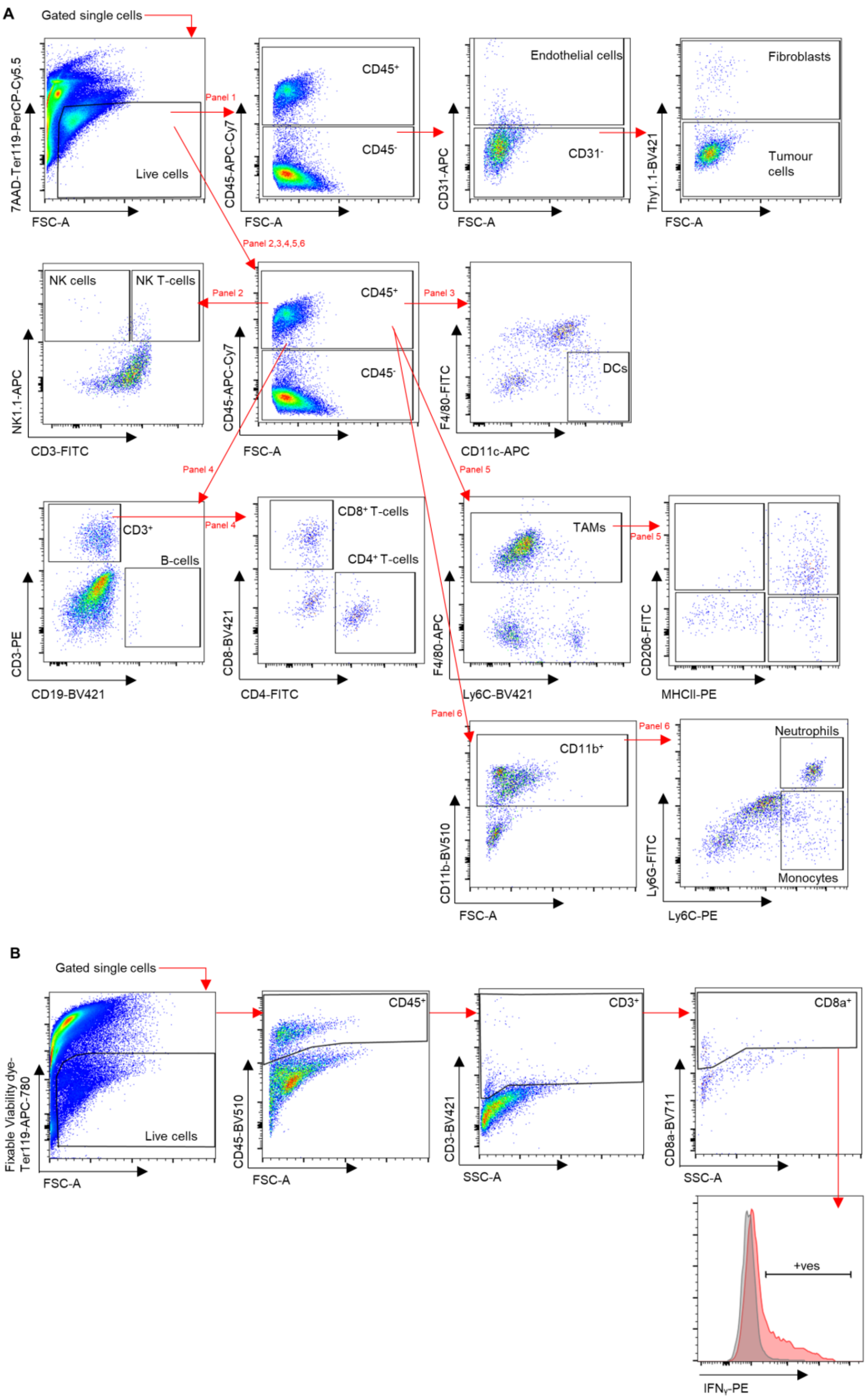

Figure S4. Flow cytometry gating strategy for tumoral populations and T-cell activation. (A-B) Representative gating strategy for identifying live nucleated tumoral populations **(A)** and IFNγ expression by T-cells **(B)** in enzyme-dispersed tumors from *MMTV-PyMT* mice using flow cytometry. Positive gates are applied based upon FMO stains.

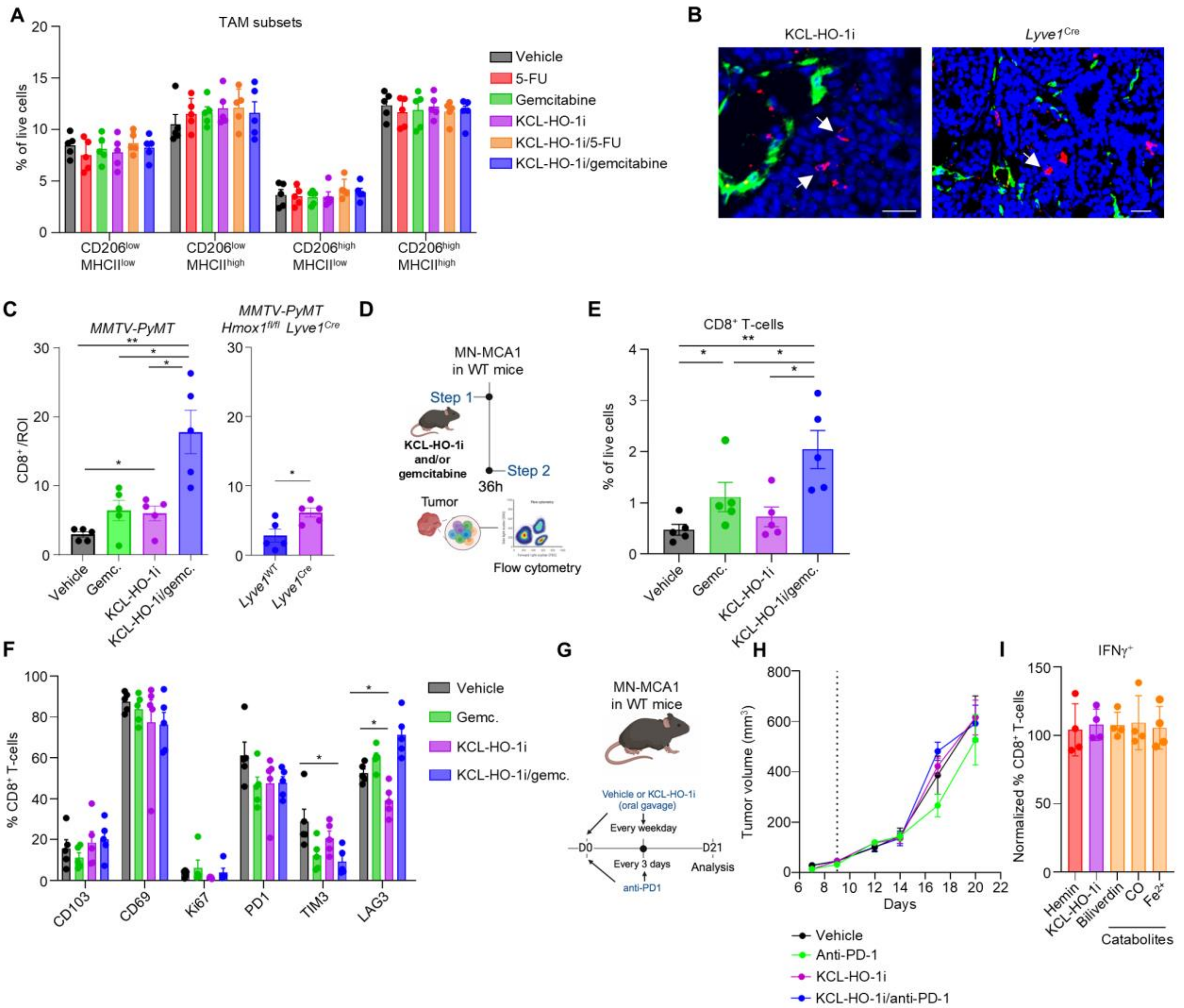

Figure S5. Characterization of the stromal response to HO-1 inhibition in murine cancer models. (A) Tumors were enzyme-digested to release single cells which were analyzed for their live (7AAD$^-$) TAM subset composition was assessed using flow cytometry (n=5 tumors per group). **(B-C)** Representative image of a frozen section of a *MMTV-PyMT* tumor treated with KCL-HO-1i (left) or from a *Lyve1*^{Cre} *Hmox1*$^{fl/fl}$ mouse (right). stained with DAPI (nuclei;blue) and antibodies against CD8 (red) and CD31 (green), Scale bar is 25 µm and white arrows indicated infiltrating CD8$^+$ T-cells **(B)** and quantification of T-cells per region of interest (ROI) for the indicated conditions and backgrounds **(C)**. **(D-F)** Schematic representing the dosing strategy where KCL-HO-1i and/or gemcitabine and/or vehicle were administered to C57Bl/6 mice bearing 500-700 mm^3 tumors. At 36 h post treatment initiation of treatment mice were sacrificed and tumors were harvested and analyzed by flow cytometry **(D)** and the abundance of CD8$^+$ T-cells **(E)** and their expression of the indicated activation and exhaustion markers **(F)** were quantified. **(G-H)** Schematic representing the dosing strategy for i.p. dosing strategy for anti-PD-1 antibodies (12 mg/kg/3 days and/or KCL-HO-1i (25 µMol/kg/day) and/or vehicle **(G)** and growth curves of established MN-MCA1 tumors under the indicated treatments **(H)**, dashed line indicates the start of treatment (cohorts of n=6 mice). **(I)** *In vitro* stimulated murine CD8$^+$ T-cells exposed to the indicated conditions for 24 h prior to evaluating their ability to secrete IFNγ post *ex vivo* exposure to PMA/ionomycin treatment using flow cytometry. Images in panel **(D)** and **(G)** were created using *BioRender*. Bar charts show the mean, error bars SD, and the dots show individual

data points from individual tumors and mice. Line charts display the mean and SEM. Gemc.; gemcitabine. * *P*<0.05, ** *P*<0.01.

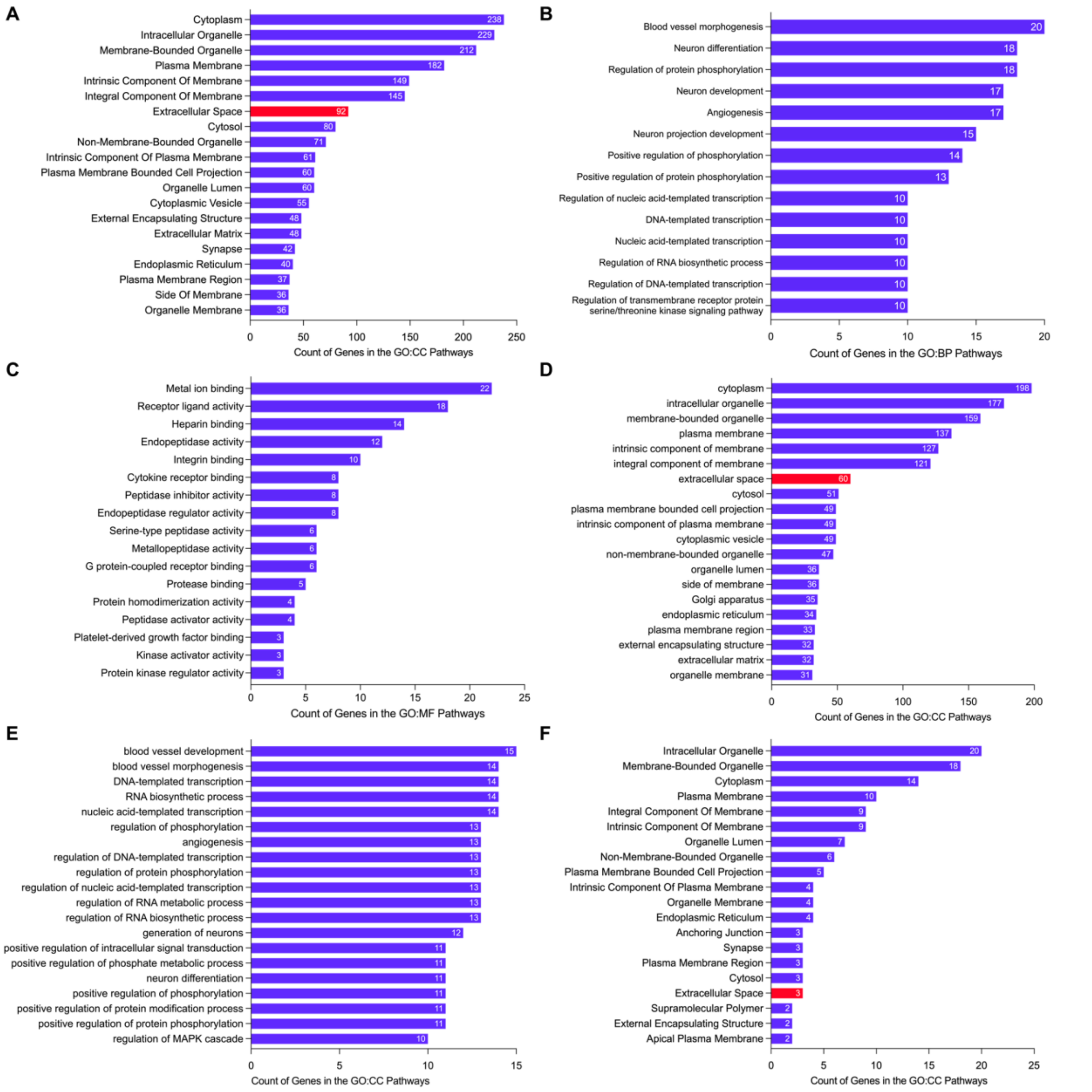

Figure S6. Cellular compartment of upregulated DEGs post treatment with KCL-HO-1i and chemotherapy. Mice bearing established *MMTV-PyMT* tumors were treated with KCL-HO-1i (25µMol/kg/day) and/or 5-FU (40 mg/kg) or gemcitabine (6.6mg/kg) or vehicle and tumor tissue were analyzed at 36 h post treatment initiation by NGS bulk RNAseq. **(A-B)** The individual cellular compartment of upregulated DEGs that are associated with KCL-HO-1i treatment (419 genes from the intercept of groups shown in Figure 5C) **(A)** and the gene ontology analysis of all DEGs for biological process (BP) **(B)** and molecular function (MF) **(C)**. **(D-E)** The individual cellular compartment of upregulated DEGs that are associated with KCL-HO-1i/chemotherapy (5-FU and gemcitabine) treatment (384 genes from the intercept of groups shown in Figure 5C) **(D)** and the gene ontology analysis of all DEGs for biological process (BP) **(E)**. **(D)** The individual cellular compartment of upregulated DEGs that are associated with chemotherapy treatments (5-FU and gemcitabine; 30 genes from the intercept of groups shown in Figure 5E).

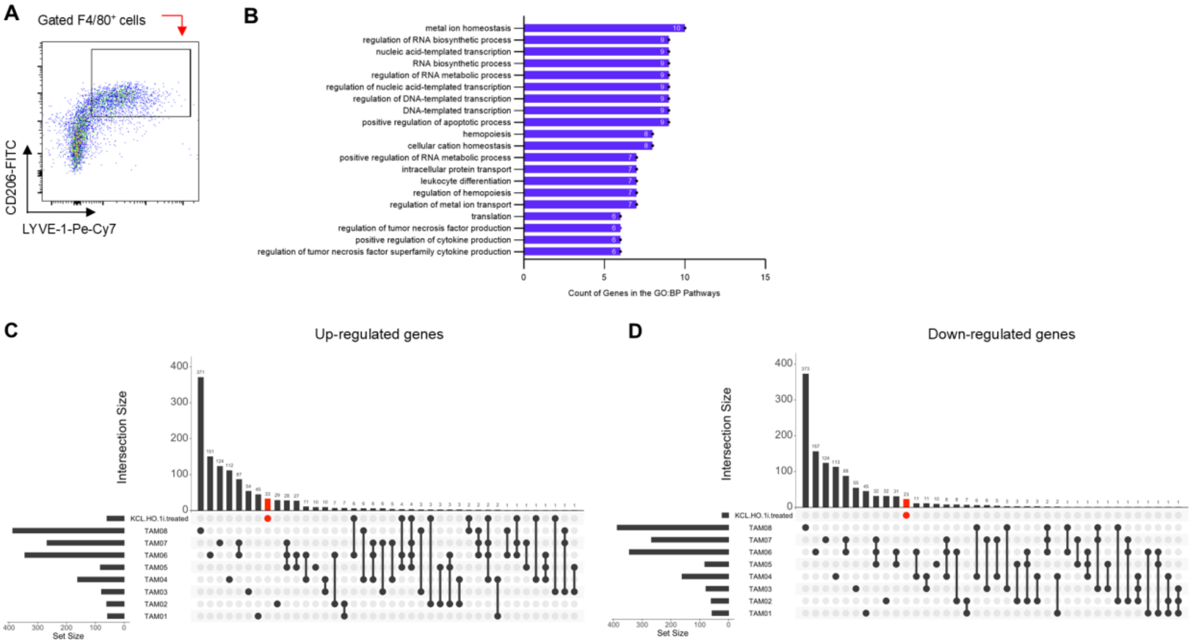

Figure S7. Differential gene expression profile of sorted LYVE1⁺TAM after KCL-HO-1i treatment in *MMTV-PyMT* mice. (A) Representative gating strategy of sorted live (7AAD⁻) CD45⁺F4/80⁺CD206⁺LYVE1⁺ TAM from *MMTV-PyMT* mice**. (B)** Gene ontology analysis of the upregulated genes after KCL-HO-1i treatment for biological process (BP). **(C-D)** Upset plot showing the overlap of differentially upregulated **(C)** and downregulated **(D)** genes between the sorted LYVE1⁺TAM population after KCL-HO-1i treatment and all the differentially upregulated genes of the eight TAM subsets described in *Opzoomer et al.*(18), where TAM 6 represents the LYVE-1⁺ population. No DEGs identified post KCL-HO-1i treatment (top line) relate to another TAM phenotype, suggesting there is no re-polarization of the PvTAM subset in response to treatment in this model.

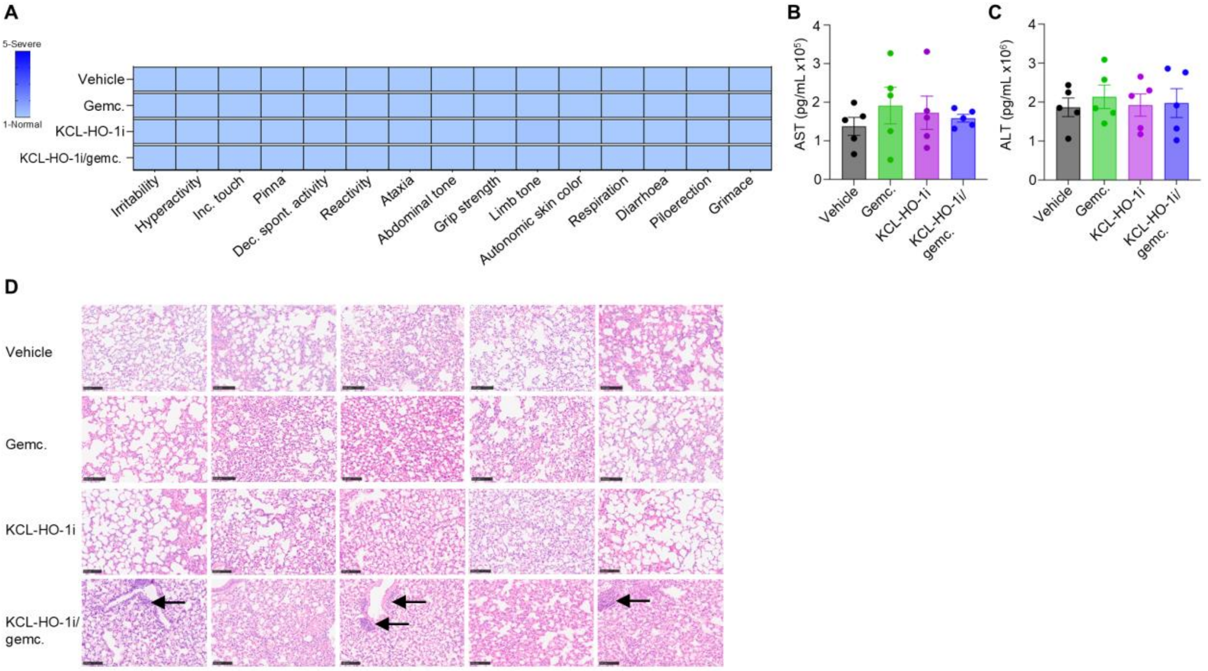

Figure S8. Oral administration of KCL-HO-1i shows low toxicity. (A-D) *MMTV-PyMT* mice bearing established tumors were treated with p.o. dosing of KCL-HO-1i (25μMol/kg/day) and/or the i.p. dosing for gemcitabine (6.6mg/kg/7days) or vehicle for 21 days (cohorts of n=5 mice). At the end of treatment mouse behavioural phenotype at the end of treatment was assessed against the parameters indicated. Scores were assessed on a grading system from 1 to 5, where 1 = normal behaviour and 5 = severely affected behaviour (mean shown across 5 mice) **(A)**. The aspartate aminotransferase (AST) **(B)** and alanine transferase (ALT) **(C)** were evaluated in the mice plasma by ELISA and absolute quantifications were shown. Representative H&E-stained tissue sections of the lungs for the different treatment groups, scale bar is 100 μm **(D)**. Bar charts show the mean, error bars SD, and the dots show individual data points from individual mice. Gemc.; gemcitabine.
